# Methodological quality and reporting quality of COVID-19 living systematic review: a cross-sectional study

**DOI:** 10.1186/s12874-023-01980-y

**Published:** 2023-07-31

**Authors:** Jiefeng Luo, Zhe Chen, Dan Liu, Hailong Li, Siyi He, Linan Zeng, Mengting Yang, Zheng Liu, Xue Xiao, Lingli Zhang

**Affiliations:** 1 Department of Pharmacy, West China Second University Hospital, Sichuan University, Chengdu, China; 2 Evidence-Based Pharmacy Center, West China Second University Hospital, Sichuan University, Chengdu, China; 3 NMPA Key Laboratory for Technical Research On Drug Products In Vitro and In Vivo Correlation, Chengdu, China; 4 Key Laboratory of Birth Defects and Related Diseases of Women and Children, Ministry of Education, Sichuan University, Chengdu, China; 5 West China School of Pharmacy, Sichuan University, Chengdu, China; 6 West China School of Medicine, Sichuan University, Chengdu, China; 7 Department of Gynecology and Obstetrics, West China Second University Hospital, Sichuan University, Chengdu, China

**Keywords:** Living systematic review, Coronavirus disease 2019, Systematic review, Methodological quality, Reporting quality

## Abstract

**Objectives:**

The main objective of this study is to evaluate the methodological quality and reporting quality of living systematic reviews (LSRs) on Coronavirus disease 2019 (COVID-19), while the secondary objective is to investigate potential factors that may influence the overall quality of COVID-19 LSRs.

**Methods:**

Six representative databases, including Medline, Excerpta Medica Database (Embase), Cochrane Library, China national knowledge infrastructure (CNKI), Wanfang Database, and China Science, Technology Journal Database (VIP) were systematically searched for COVID-19 LSRs. Two authors independently screened articles, extracted data, and then assessed the methodological and reporting quality of COVID-19 LSRs using the "A Measurement Tool to Assess systematic Reviews-2" (AMSTAR-2) tool and "Preferred Reporting Items for Systematic reviews and Meta-Analyses" (PRISMA) 2020 statement, respectively. Univariate linear regression and multivariate linear regression were used to explore eight potential factors that might affect the methodological quality and reporting quality of COVID-19 LSRs.

**Results:**

A total of 64 COVID-19 LSRs were included. The AMSTAR-2 evaluation results revealed that the number of "yes" responses for each COVID-19 LSR was 13 ± 2.68 (mean ± standard deviation). Among them, 21.9% COVID-19 LSRs were rated as "high", 4.7% as "moderate", 23.4% as "low", and 50% as "critically low". The evaluation results of the PRISMA 2020 statement showed that the sections with poor adherence were methods, results and other information. The number of "yes" responses for each COVID-19 LSR was 21 ± 4.18 (mean ± standard deviation). The number of included studies and registration are associated with better methodological quality; the number of included studies and funding are associated with better reporting quality.

**Conclusions:**

Improvement is needed in the methodological and reporting quality of COVID-19 LSRs. Researchers conducting COVID-19 LSRs should take note of the quality-related factors identified in this study to generate evidence-based evidence of higher quality.

**Supplementary Information:**

The online version contains supplementary material available at 10.1186/s12874-023-01980-y.

## What is already known on this topic?

Despite an increasing number of COVID-19 LSRs being published, there have been no studies to assess their methodological and reporting quality, which are crucial for informing clinical practice and policy-making

## What this study adds?

Our study aimed to evaluate the methodological and reporting quality of published COVID-19 LSRs and to identify factors that could affect their quality.

## What is the implication?

Low-quality COVID-19 LSRs may undermine the confidence of clinicians and policymakers in the evidence, thereby hindering its translation into practice. This study serves as a reference for future researches of COVID-19 LSRs.

## Introduction

The 2019 Coronavirus Disease (COVID-19) pandemic is still a major public health problem on a global scale. Subsequent evidence has confirmed that it’s caused by a novel coronavirus, initially referred to as 2019-novel coronavirus (2019-nCoV) by the World Health Organization (WHO) [1]. The WHO declared the COVID-19 outbreak a pandemic on February 11, 2020 [2]. As of September 19, 2022, there have been 610,393,563 confirmed cases of COVID-19 and 6,508,521 deaths reported to WHO [3]. Researchers worldwide are working diligently to understand COVID-19 as soon as possible. However, amidst the massive amount of published evidence, "false information" and "false conclusions" have emerged, forming an "Infodemic" that can increase clinician's workload and hinder problem-solving efforts [4–6].

Systematic reviews (SRs) and meta-analysis (MAs) are the results of a rigorous scientific process consisting of several well-defined steps, including a systematic literature search, an evaluation of the quality of each included study and a quantified or narrative synthesis of the results obtained [7]. SRs and MAs are often considered as the highest level of evidence in evidence-based medicine, as they can bridg the gap between clinical research and clinical practice [8, 9]. Healthcare decision-makers in search of reliable information increasingly turn to SRs for the best summary of the evidence [10, 11]. However, traditional SRs are not updated or updated at long intervals (Cochrane SRs require updates every two years), which is inadequate for the rapidly evolving COVID-19 pandemic [12, 13]. Inability to maintain currency under the COVID-19 pandemic may lead to significant inaccuracy [14].

Living systematic reviews (LSRs), proposed by Julian and his colleagues in 2014, are a unique type of SRs that are continually updated as new evidence becomes available [14, 15]. Studies under the COVID-19 pandemic meet exactly three conditions for conducting LSR: (1) The review question is a particular priority for decision-making; (2) There is an important level of uncertainty in the existing evidence; (3) There is likely to be emerging evidence that will impact on the conclusions of the LSR [14, 16]. Therefore, LSRs have become increasingly important under the COVID-19 pandemic.

Well-conducted SRs provide an excellent snapshot of evidence [17], conversely, poor methodological and reporting quality may reduce the confidence of clinicians and policymakers in the conclusions of SRs [18, 19]. Therefore, it is necessary to assess the quality of SRs before applying their conclusions to clinical or public health practice [20]. As a unique kind of SRs, the same holds true for LSRs, which are even more important under the COVID-19 pandemic. Although there have been studies assessing the methodological and reporting quality of COVID-19 SRs [21–23], to our knowledge, none have yet evaluated the quality of COVID-19 LSRs.

Therefore, the main objective of this study is to evaluate the methodological quality and reporting quality of LSRs on COVID-19, while the secondary objective is to investigate potential factors that may influence the overall quality of COVID-19 LSRs. The findings of this study will provide useful insights for the development of future COVID-19 LSRs.

## Methods

A cross-sectional study was conducted. In this study, it is important to note that given the continued spread of COVID-19 and the rapid development of LSR, we did not have a published protocol prior to conducting this study. This study was reported according to the Strengthening the Reporting of Observational studies in Epidemiology (STROBE) guidelines [24]. (Supplementary material Appendix I).

### Search strategy

Six databases including Medline, Excerpta Medica Database (Embase), Cochrane Library, China national knowledge infrastructure (CNKI), Wanfang Database and China Science, Technology Journal Database (VIP) were systematically searched. We searched the databases from their inception until December 9, 2021, and additional searches were conducted on May 13, 2022. The primary search terms included living systematic review, living meta-analysis, etc. (Supplementary material Appendix II). The sample size for this study was all eligible studies.

Given that preprints are not peer-reviewed and results may still change, we did not search preprint databases [25]. We acknowledge that COVID-19 LSRs may have multiple versions due to regular updates, hence we give priority to the version that provides more information.

### Inclusion and exclusion criteria

Inclusion criteria: (1) The study type is a SR; (2) The title or abstract clearly identifies it as “living systematic reviews” (using this or similar terminology); (3) The clinical topic of systematic reviews is related to COVID-19.

Exclusion criteria: (1) Unavailable articles; (2) Withdrawn COVID-19 LSRs; (3) Living evidence map.

### Selection and information extraction

The retrieved articles were imported into ENDNOTES X8 (Thomson Corporation, Thomson ResearchSoft, USA) software for removing duplicates and selection. The review authors (Jiefeng Luo and Zheng Liu) independently screened articles in duplicate, with any disagreements resolved by a third author (Zhe Chen). The article selection process involved several steps: first, we screened out obviously irrelevant articles based on their titles and abstracts; then, we assessed the remaining articles by reading their full texts.

The data extraction was conducted independently and in duplicate by the review authors (Jiefeng Luo and Zheng Liu), with any disagreements being resolved by a third author (Zhe Chen). The data extraction form was designed in advance based on the pre-extracted data from ten COVID-19 LSRs. The data extraction form included title, first author, year of publication, country and region of publication, journal of publication and eight factors that might affect overall quality of COVID-19 LSRs. These factors include impact factor (IF), number of authors, number of institutions, number of included studies, whether there were international collaborations (yes or no), whether authors stated their funding sources (yes or no), whether the study was pre-registered in any registration platform (yes or no), and whether authors reported compliance with the PRISMA statement (yes or no).

### Methodological quality assessment

The methodological quality of COVID-19 LSRs was assessed independently and in duplicate by the review authors (Jiefeng Luo and Zheng Liu), with any disagreements resolved by a third author (Zhe Chen). Although there are numerous tools available for assessing the methodological quality of SRs, we opted for AMSTAR-2 due to its widespread usage and established validity and reliability [19, 26, 27]. AMSTAR-2 consists of 16 domains, of which Domain 2, Domain 4, Domain 7, Domain 9, Domain 11, Domain 13, and Domain 15 are critical domains. Answers for each domain include three options: "yes", "partial yes", and "no". The methodological quality of SRs was divided into four levels according to the following criteria: high (No or one non-critical weakness), moderate (More than one non-critical weakness), low (One critical flaw with or without non-critical weaknesses) and critically low (More than one critical flaw with or without non-critical weaknesses). It is worth noting that Domain 11, 12 and 15 would no longer apply if no meta-analysis has been performed. Considering that multiple non-critical weaknesses may reduce confidence in the review, we defined LSR with more than 4 non-critical weaknesses as "Low".

### Reporting quality assessment

The reporting quality of COVID-19 LSRs was assessed independently and in duplicate by the review authors (Jiefeng Luo and Zheng Liu), with any disagreements resolved by a third author (Zhe Chen). PRISMA statement was used to assess the reporting quality of included COVID-19 LSRs. PRISMA is aimed to guide SRs for complete reporting and to improve the transparency and reporting quality of SRs [18]. While there are various PRISMA statement extensions available to facilitate reporting on different types or aspects of SRs, we have chosen to use the PRISMA 2020 statement as the assessment tool for reporting quality of COVID-19 LSRs [28]. This decision was made because we recognized that different versions of the PRISMA statement might result in incomparable items.

PRISMA 2020 statement includes seven sections (title, abstract, introduction, methods, results, discussions and other information) with 27 items, and each item was assessed as "yes", "partial yes", or "no" based on the degree of compliance with the reporting criteria. We calculated the number of "yes" responses for each COVID-19 LSR and defined that the larger the number of "yes" responses, the better the reporting quality of the COVID-19 LSRs.

### Statistical analysis

EXCEL 2019 (Microsoft Corporation, WA, USA) was used to quantitatively analyze and qualitatively describe the included COVID-19 LSRs. For all categorical variables such as AMSTAR-2 levels, international collaborations (yes or no), funding (yes or no), pre-registration (yes or no), and PRISMA statement (yes or no), we used frequencies and percentages. For all continuous variables, including the number of "yes" responses of PRISMA 2020 statement, the number of "yes" responses of AMSTAR-2, IF, number of institutions, number of authors, and number of included studies, we used mean, median, standard deviation (SD), and range.

To investigate factors that could potentially affect the methodological quality and reporting quality in COVID-19 LSRs, we conducted linear regression analysis on eight factors as described above. We conducted the linear regression analysis in two steps: firstly, we performed univariate linear regression on the eight factors, and subsequently, we performed multiple linear regression on those factors with statistical differences. We defined factors with statistical differences in multiple linear regression as determinants of quality [29]. We used the variance inflation factor (VIF) to assess multicollinearity among study features, and a VIF ≥ 5 was considered highly correlated [30].

## Results

### Study selection

**Fig. 1:**
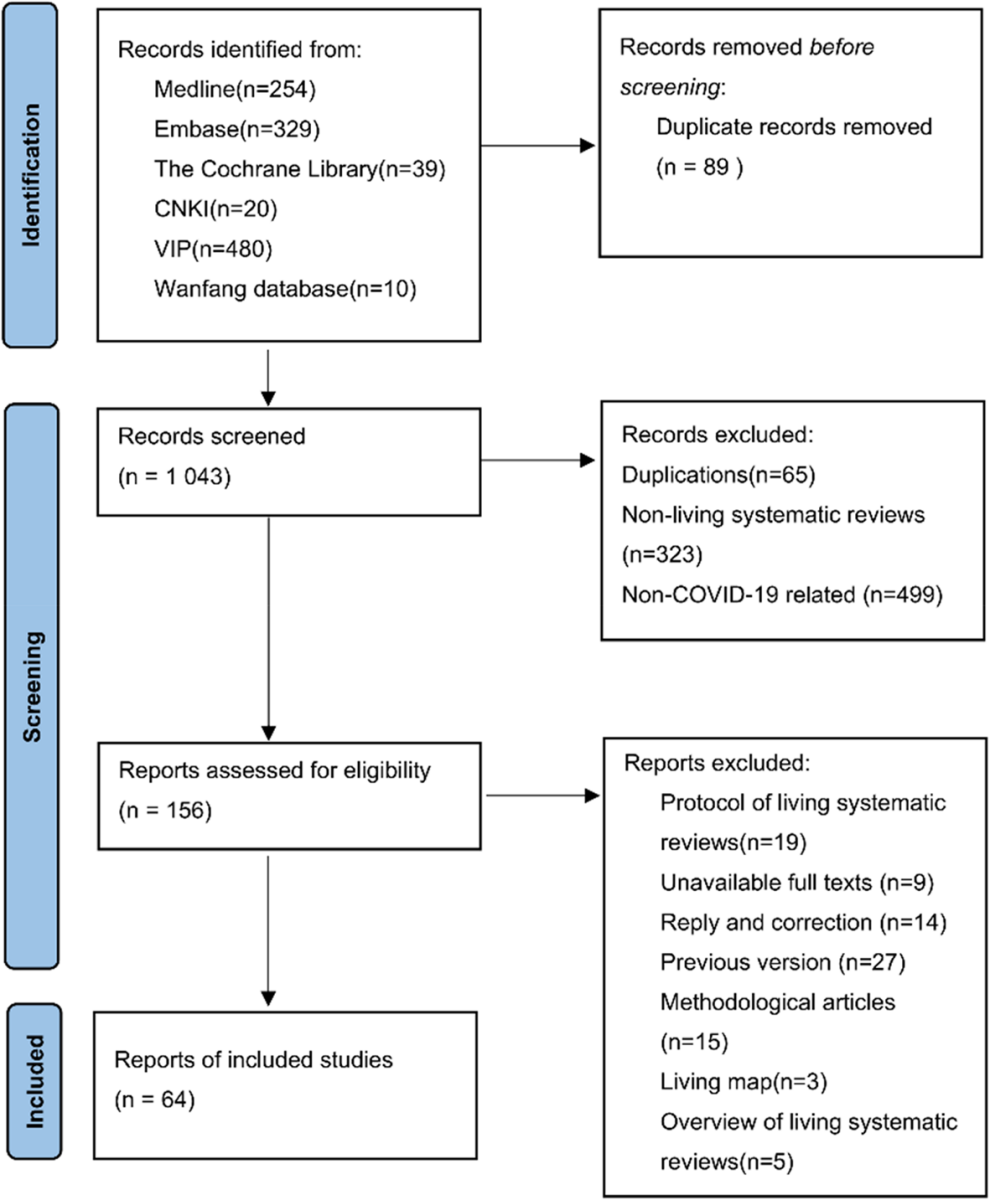
Flow diagram of screening

A total of 1,132 articles were initially included, and 1,043 articles remained after removing duplicate articles by ENDNOTES X8. Then 156 articles remained after excluding obviously irrelevant articles by screening the title and abstract. And finally, 64 COVID-19 LSRs were included by reading the full text [31–94]. The flow diagram of the screening process is presented in Fig. 1. The titles and reasons for excluded studies are presented in Appendix VI of the Supplementary Material.

### Study characteristics

Table 1 summarizes the characteristics of 64 COVID-19 LSRs we included, with additional details available in Appendix III-V. Most COVID-19 LSRs were published in high-impact Science Citation Index (SCI) journals, with 23% having an IF greater than 10. LSRs involved multiple institutions and authors, with an average of 9.27 institutions and 14.53 authors per LSR. The number of included studies in LSRs ranged from 0 [37, 73]to 728 [67]. Many COVID-19 LSRs followed the PRISMA statement (78.1%), involved collaboration across multiple countries (59.4%), and were funded (81.3%). Additionally, a majority of the COVID-19 LSRs were registered (81.3%).

**Table 1:** Study characteristics

Category	Mean, (Median and Range)	Characteristic	Number	Percentage *N* = 64
IF	11.72(8.06, 0–39.89)	Non-SCI	7	10.9%
		IF ≤ 5	16	25.0%
		5 < IF ≤ 10	26	40.6%
		10 < IF ≤ 15	4	6.3%
		IF ≥ 15	11	17.2%
Number of institutions	9.27(7, 1–37)	Single institution	2	3.1%
		1 < Number of institutions ≤ 5	19	29.7%
		5 < Number of institutions ≤ 10	22	34.4%
		10 < Number of institutions ≤ 15	11	17.2%
		15 < Number of institutions	10	15.6%
Number of authors	14.53(2–57)	Single author	0	0%
		1 < Number of authors ≤ 5	9	14.1%
		5 < Number of authors ≤ 10	26	40.6%
		10 < Number of authors ≤ 15	10	15.6%
		15 < Number of authors	19	29.7%
Number of included studies	57.36(17.5, 0–728)	Number of included studies = 0	2	3.1%
		0 < Number of included studies ≤ 10	21	32.8%
		10 < Number of included studies ≤ 20	10	15.6%
		20 < Number of included studies ≤ 50	14	21.9%
		50 < Number of included studies ≤ 100	9	14.1%
		100 < Number of included studies	8	12.5%
Follow the PRISMA statement	/	Yes	50	78.1%
		No/Unclear	14	21.9%
International collaborations	/	Yes	38	59.4%
		No	26	40.6%
Funding	/	Yes	52	81.3%
		No/Unclear	12	18.8%
Registration	/	Yes	52	81.3%
		No/Unclear	12	18.8%

### Methodological quality

**Fig. 2:**
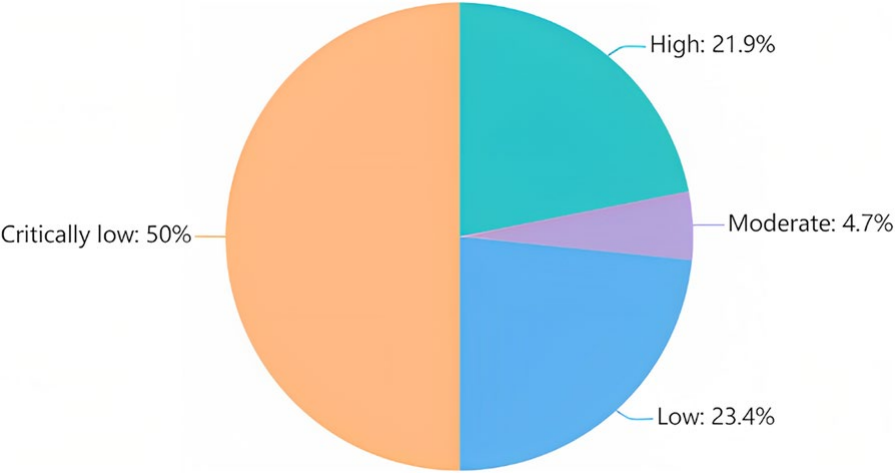
AMSTAR-2 levels distribution

The evaluation results of the 64 COVID-19 LSRs based on the AMSTAR-2 had an average of 13 "yes" responses, with a median of 11, a range between 6 and 16, and a standard deviation of 2.68. 50% COVID-19 LSRs were assessed "critically low", 23.4% were "low", 4.7% were "moderate", and only 21.9% were "high". Figure 2 displays the distribution of methodological quality levels.

**Fig. 3:**
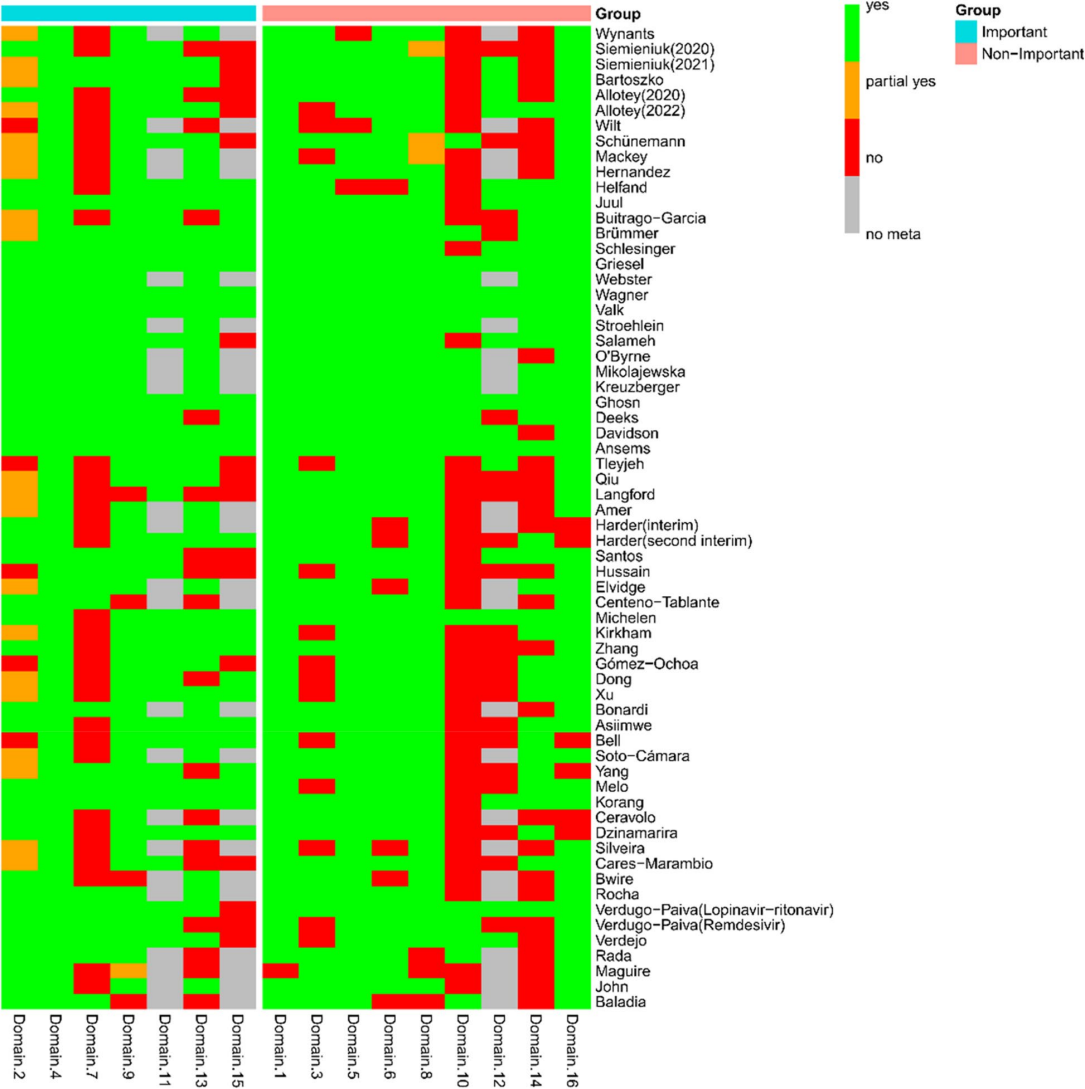
Heat map of AMSTAR-2

Domain 1, Domain 4, Domain 5, Domain 8, Domain 9, Domain 11 and Domain 16 were reported by more than 90% of COVID-19 LSRs. The worst methodological quality is Domain 10, with only 31.25% of COVID-19 LSRs reports. Figure 3 shows a heatmap of the assessment results of each domain in the 64 COVID-19 LSRs included by AMSTAR-2. From the figure, it is clear that LSRs adherence to critical domains was better than that of non-critical domains.

### Reporting quality

**Fig. 4:**
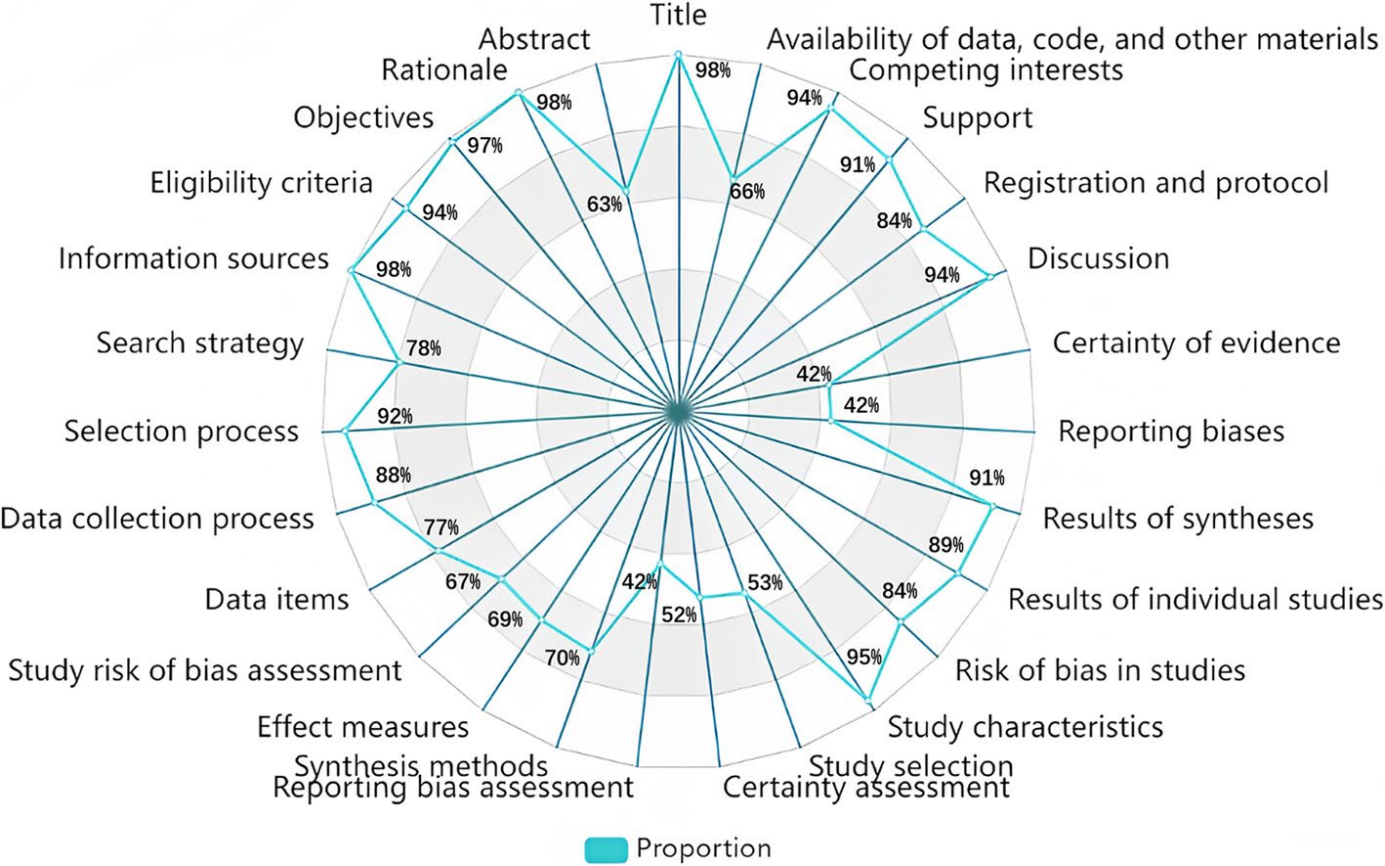
Proportion of “yes” for each item in PRISMA 2020 Statement

The evaluation results of the 64 COVID-19 LSRs based on the PRISMA 2020 statement had an average of 21 "yes" responses, with a median of 21, a range between 13 and 27, and a standard deviation of 4.18. Figure 4 displays the PRISMA evaluation results for each item of the COVID-19 LSRs, presented as the percentage of "yes" responses.

**Fig. 5:**
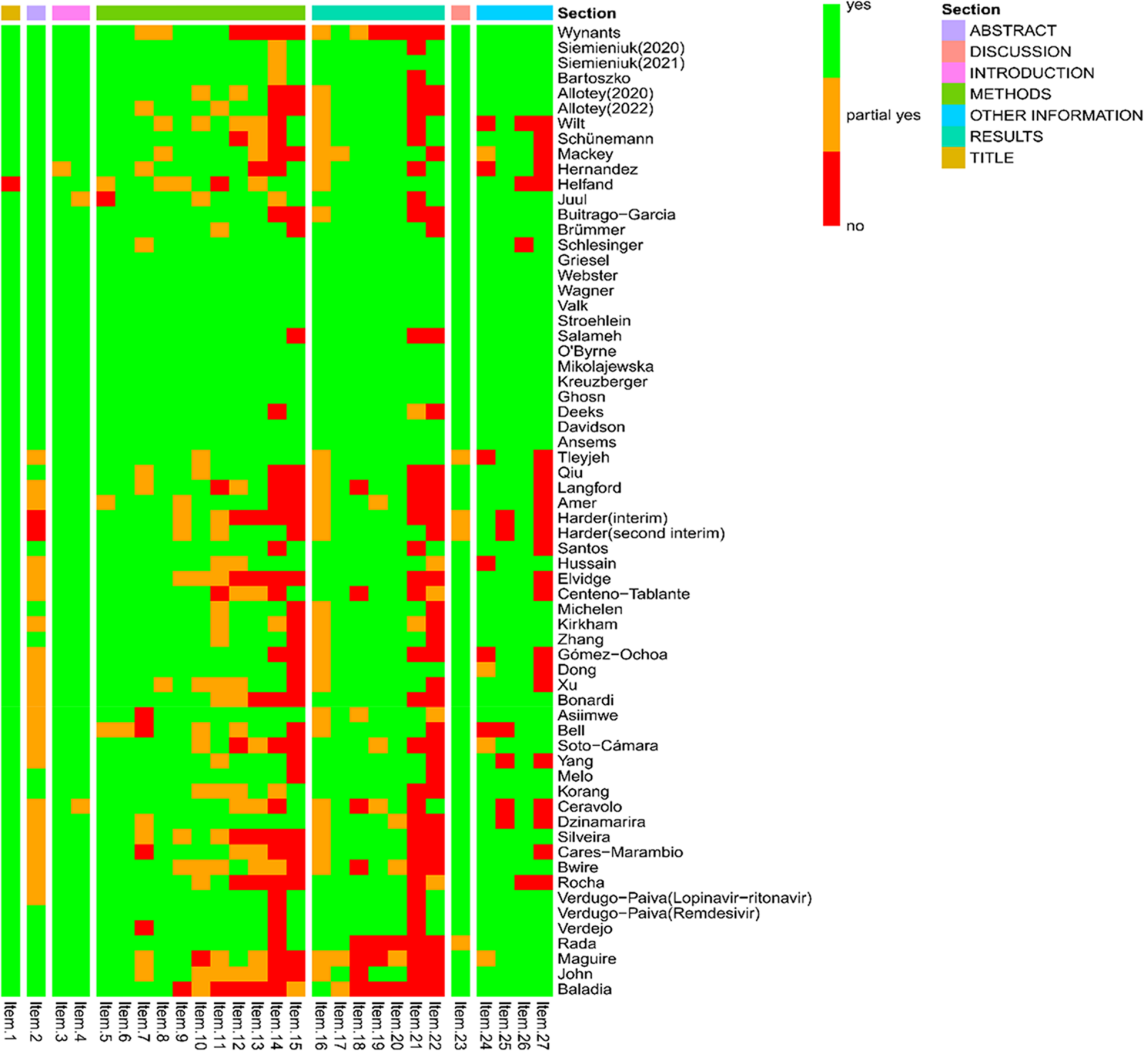
Heat map of PRISMA 2020 statement

More than 90% of the COVID-19 LSRs fully reported 9 items (Item 1, Item 3, Item 4, Item 5, Item 6, Item 8, Item 17, Item 23, and Item 26), whereas less than 50% fully reported 5 items (Item 14, Item 15, Item 16, Item 21, and Item 22). The "Title", "Rationale", "Objectives", and "Information sources" had the best reporting quality, with 98% of COVID-19 LSRs fully reporting them. On the other hand, the "Certainty of evidence" had the worst reporting quality, with only 41% of COVID-19 LSRs fully reporting it. Figure 5 shows a heatmap of the assessment results of each item in the 64 COVID-19 LSRs included by PRISMA 2020 statement. From the figure, it is clear that the sections with poor adherence were methods, results and other information.

### Results of correlation analyses

The results of univariate and multivariate analyses of the correlation between the eight factors and the overall quality of COVID-19 LSRs are shown in Table 2.

**Table 2:** Linear regression results of PRISMA statement and AMSTAR-2

Category	Number of "yes" in PRISMA/AMSTAR-2 rating	Univariate analysis	Multivariate analysis		
		Coefficient (95% CI)	Coefficient (95% CI)	Adjusted R^2^	VIF
IF	Number of "yes" in PRISMA	-0.050,0.135			
	AMSTAR-2 rating	-0.047,0.005			
Number of authors	Number of "yes" in PRISMA	-0.057,0.118			
	AMSTAR-2 rating	-0.035,0.015			
Number of institutions	Number of "yes" in PRISMA	-0.076,0.205			
	AMSTAR-2 rating	-0.038,0.043			
Number of included studies	Number of "yes" in PRISMA	**-0.018**,**0.000**	**-0.019**,**-0.001**	**0.142**^a^	**1.013**
	AMSTAR-2 rating	**-0.006**,**0.000**	**-0.006**,**-0.001**	**0.192**^**b**^	**1.001**
International collaborations	Number of "yes" in PRISMA	-0.141,4.031			
	AMSTAR-2 rating	-0.447,0.783			
Funding	Number of "yes" in PRISMA	**0.710**,**5.842**	**1.105**,**6.095**	**0.142 **^a^	**1.013**
	AMSTAR-2	-0.272,1.259			
Registration	Number of "yes" in PRISMA	-2.395,2.997			
	AMSTAR-2 rating	**0.386**,**1.832**	**0.448**,**1.832**	**0.192 **^b^	**1.001**
PRISMA statement	Number of "yes" in PRISMA	-0.857,4.166			
	AMSTAR-2 rating	-0.843,0.620			

*CI* Confidence interval, *VIF* Variance inflation factor

Bolded parts indicate that they are statistically different

^a^ Number of included studies" and "Funding" explained for 14.2% of the variation in "Number of ' yes' in PRISMA"

^b^ Number of included studies" and " Registration" explained for 19.2% of the variation in "AMSTAR-2 rating"

The Table 2 showed that the number of included studies, and registration are associated with AMSTAR-2 levels, and these variables explained a total of 19.2% of the variation in AMSTAR-2 levels; the number of included studies and funding are associated with the number of “yes" in PRISMA 2020 statement, and these variables explained a total of 14.2% of the variation in the number of “yes" in PRISMA 2020 statement.

## Discussion

The concept of LSR was proposed by Julian and his colleagues more than nine years ago, but previous studies on LSR were tepid until the outbreak of COVID-19, which triggered a surge in related research [95]. At present, the research methods of LSR are still under exploration. Therefore, it is of great significance to summarize and analyze the existing LSRs quality, and determine potential influencing factors. To our knowledge, this is the first study to assess the quality of COVID-19 LSRs and attempt to identify potential influencing factors. We believe that this is crucial in guiding the implementation of future LSRs under COVID-19.

The methodological quality of 73.4% of COVID-19 LSRs has been assessed as low or critically low. In Domains 10 and Domains 12, the compliance rate were below 50%. The content of Domain 10 is whether SR authors report funding information of the included studies, with a compliance rate of only 31.25%. Studies funded by corporations may be more biased towards the sponsor. Therefore, it is helpful for SR authors to extract and report the funding information of the included studies for readers to judge its influence on the SR. We recommend that future COVID-19 LSRs authors and journal editors adhere to the relevant requirements of Domains 10. Domains 12 assess whether SR authors used Risk of Bias (ROB) tools to evaluate the potential influence of individual studies. The compliance rates for this domain was 31.25%, indicating that a significant proportion of the COVID-19 LSRs included did not meet this criteria. When authors include RCTs of varying quality, Domain 12 becomes particularly crucial, as RCTs with a high risk of bias can distort facts and reduce the credibility of the evidence [96]. Therefore, we recommend that authors of COVID-19 LSRs employ regression analysis to assess the impact of bias on the results when including RCTs of different quality, or restrict the analysis to studies with a low risk of bias to observe the stability of the results.

On the other hand, the average of "yes" responses to PRISMA 2020 statement for each COVID-19 LSR was 21, which only accounted for 77.8% of all items, with items 14, items 21, and items 22 having compliance rates below 50%. Item 14 and Item 21 are whether the authors report "reporting biases" in the methods/results. We speculate that the reason for the low compliance rate for these two items is that in the early stages of conducting the COVID-19 LSR, the number of included studies was small, so the authors did not consider reporting biases (the Cochrane Handbook recommends using a funnel plot to test for reporting biases when including more than 10 studies). We recommend that the authors specify the method for testing "reporting biases" in the protocol before conducting the COVID-19 LSR. When the number of included studies is too small, the reason for not testing "reporting biases" should be explained in the results. Item 22 is to present assessments of the certainty or confidence in the body of evidence for each assessed outcome. Currently, the Grading of Recommendations, Assessment, Development and Evaluations (GRADE) tool is widely used to grade the quality of evidence for each outcome. Murad suggested that the GRADE rating results can be used as triggers for retiring LSRs from the living mode [97]. Therefore, we strongly recommend that authors of COVID-19 LSRs use the GRADE tool to grade the quality of evidence for each outcome.

Through linear regression, we have identified the factors that influence the quality of COVID-19 LSRs, including the number of included studies, funding, and registration (all of which are positively correlated). Given that including more studies may be associated with higher-quality COVID-19 LSRs, we recommend that authors conduct a more comprehensive search and make their utmost efforts to include all eligible studies. Existing evidence has confirmed the high academic value of gray literature during the COVID-19 pandemic [98]. Therefore, authors may consider including eligible gray literature, as appropriate, to enhance the quality of COVID-19 LSRs. Funding may mean a more diverse author team (e.g. methodologists, informaticians), and sustaining LSRs requires funding [99], so we suggest that authors should obtain as much funding as possible for their research. Many studies have shown that registration is positively correlated with SR’s quality [100–102], as it helps to avoid authors selectively reporting findings that favor publication. Therefore, we recommend that all authors of COVID-19 LSRs register and present their protocol on websites/journals.

Surprisingly, claiming adherence to the PRISMA statement did not improve the reporting quality of COVID-19 LSRs. We speculate that this may be due to almost half of the LSRs (46.9%) following the PRISMA 2009 statement (as the PRISMA 2020 statement was released in March 2021), which has significant differences from the PRISMA 2020 statement.

The potential association among international cooperation, number of authors, and number of institutions (i.e., more authors means more institutions, which necessarily implies more international collaboration) may be one of the reasons why they do not show a correlation with the quality of COVID-19 LSRs.

Similar to Zheng's findings [95], our study found that over 89% of COVID-19 LSRs were published in SCI journals (This figure was 76.8% in Zheng's findings), with more than 64% of these journals having an IF greater than 5. This reflects the importance that high-impact journals place on COVID-19 LSRs and facilitates the wide dissemination of evidence [22]. In Zheng's findings, over 97% of LSRs were published in English. Similarly, in this study, all the COVID-19 LSRs were published in English. This suggests that LSR authors prioritize international communication of their findings, which could facilitate overcoming language barriers in translating clinical evidence into practice across different countries.

**Fig. 6:**
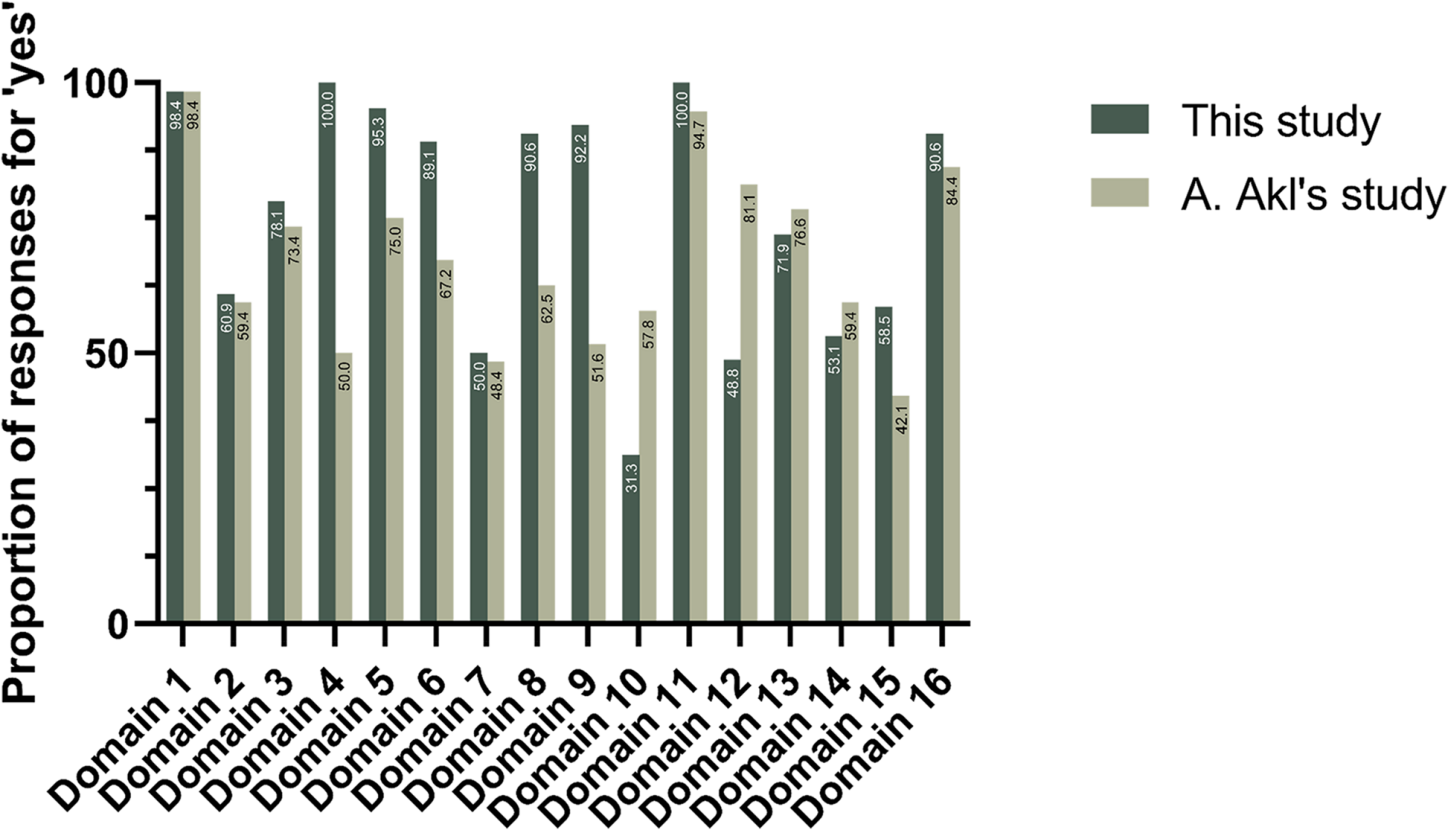
Comparison of the proportion of "yes" in each domain between this study and A. Akl's study for AMSTAR-2

Despite conducting a comprehensive search, we did not identify any studies evaluating the quality of COVID-19 LSRs. Only one study, published in 2023, evaluating the quality of different versions of LSRs was included for comparison in this study. A. Akl and his colleagues assessed the methodological and reporting quality of 64 LSRs (base version) published from February 2013 to April 2021 using AMSTAR-2 and the PRISMA 2009 statement, respectively [103]. The methodological quality of the two studies was generally consistent, except for domains 4, 8, 9,10 and 12. A comparison of the proportion of "yes" in each domain between this study and A. Akl's study for AMSTAR-2 is presented in Fig. 6. It is our speculation that the reason behind these variations is the significant differences in the inter-rater reliability of AMSTAR, which is influenced by the pairing of reviewers. Additionally, A. Akl's study only included 63% of LSRs that are COVID-19 related. Due to the incomparability between PRISMA 2009 statement and PRISMA 2020 statement, we did not compare the differences in reporting quality between this study and A. Akl's study. Unfortunately, the primary objective of A. Akl's study was not to evaluate the quality of LSRs but rather to describe their characteristics and understand their life cycles. Consequently, several important data points, such as the methodological and reporting quality results for each individual LSR, were not accessible. Therefore, A. Akl's study does not provide prescriptive guidance for LSR’s authors. In contrast, our study focuses specifically on COVID-19-related LSRs and the findings can offer valuable insights for future authors of COVID-19 LSRs.

### Strengths and limitations

Our study has several advantages. Firstly, we believe that we are the first to evaluate the methodological and reporting quality of COVID-19 LSRs. Secondly, our study identifies potential factors that could impact the quality of COVID-19 LSRs, which could inform future development of such studies. Thirdly, we conducted a systematic search, including an updated search in May 2022, to ensure all eligible COVID-19 LSRs were included. However, our study also has limitations. Firstly, the PRISMA 2020 statement acknowledges that applying it to LSRs presents some challenges, such as reporting key data during the production process (e.g., search frequency, screening frequency, update frequency). Secondly, we used the AMSTAR-2 tool to assess all included COVID-19 LSRs, but this tool is designed for assessing healthcare intervention SRs and is not suitable for evaluating the "living" domain in the production of COVID-19 LSRs.

## Conclusion

Improvement is needed in the methodological and reporting quality of COVID-19 LSRs. Researchers conducting COVID-19 LSRs should take note of the quality-related factors identified in this study to generate evidence-based evidence of higher quality.

## Supplementary Information

**Additional file 1: Appendix I.** STROBE Checklists.** Appendix II.** SEARCH STRATEGIES.** Appendix III.** PRISMA statement 2020 assessment results.** Appendix IV.** AMSTAR-2 assessment results.** Appendix V.** Characteristics of the included studies.** Appendix VI.** List of the excluded studies.

## Data Availability

The data used to support the findings of this study are included within the supplementary information.

## References

[1] Lu H, Stratton CW, Tang YW (2020). Outbreak of pneumonia of unknown etiology in Wuhan, China: The mystery and the miracle. J Med Virol.

[2] WHO[homepage on the Internet]. Naming the coronavirus disease (COVID-19) and the virus that causes it. 2020–02–11; Available from:https://www.who.int/emergencies/diseases/novel-coronavirus-2019/technical-guidance/naming-the-coronavirus-disease-(covid-2019)-and-the-virus-that-causes-it, 2022–05–09.

[3] World Health Organization[homepage on the Internet]. WHO Coronavirus Disease (COVID-19) Dashboard. 2022; Available from:https://covid19.who.int/. Accessed 03–09, 2022.

[4] Zarocostas J (2020). How to fight an infodemic. Lancet (London, England).

[5] Casigliani V, De Nard F, De Vita E (2020). Too much information, too little evidence: is waste in research fuelling the covid-19 infodemic?. BMJ.

[6] Gazendam A, Ekhtiari S, Wong E (2020). The "Infodemic" of journal publication associated with the novel coronavirus disease. J Bone Joint Surg Am.

[7] The Cochrane Library[homepage on the Internet]. Systematic Reviews. Available from:https://swiss.cochrane.org/resources/systematic-reviews, 2022–05–11.

[8] Caldwell PH, Bennett T (2020). Easy guide to conducting a systematic review. J Paediatr Child Health.

[9] Wormald R, Evans J (2018). What makes systematic reviews systematic and why are they the highest level of evidence?. Ophthalmic Epidemiol.

[10] Io M (2011). Finding What Works in Health Care: Standards for Systematic Reviews.

[11] Bero LA, Jadad AR (1997). How consumers and policymakers can use systematic reviews for decision making. Ann Intern Med.

[12] Garner P, Hopewell S, Chandler J (2016). When and how to update systematic reviews: consensus and checklist. BMJ.

[13] Jadad AR, Cook DJ, Jones A (1998). Methodology and reports of systematic reviews and meta-analyses: a comparison of Cochrane reviews with articles published in paper-based journals. JAMA.

[14] Elliott JH, Turner T, Clavisi O (2014). Living systematic reviews: an emerging opportunity to narrow the evidence-practice gap. PLoS Med.

[15] Elliott JH, Synnot A, Turner T (2017). Living systematic review: 1. Introduction-the why, what, when, and how. J Clin Epidemiol.

[16] collaboration C[homepage on the Internet]. Guidance for the production and publication of Cochrane living systematic reviews: Cochrane Reviews in living mode. 2019; Available from:https://community.cochrane.org/sites/default/files/uploads/inline-files/Transform/201912_LSR_Revised_Guidance.pdf. Accessed 03–09, 2022.

[17] D'Souza R, Malhamé I, Shah PS (2022). Evaluating perinatal outcomes during a pandemic: a role for living systematic reviews. Acta Obstet Gynecol Scand.

[18] Liberati A, Altman DG, Tetzlaff J (2009). The PRISMA statement for reporting systematic reviews and meta-analyses of studies that evaluate healthcare interventions: explanation and elaboration. BMJ.

[19] Shea BJ, Reeves BC, Wells G (2017). AMSTAR 2: a critical appraisal tool for systematic reviews that include randomised or non-randomised studies of healthcare interventions, or both. BMJ.

[20] Shea BJ, Bouter LM, Peterson J (2007). External validation of a measurement tool to assess systematic reviews (AMSTAR). PLoS ONE.

[21] do Borges Nascimento IJ, O'Mathúna DP, von Groote TC (2021). Coronavirus disease (COVID-19) pandemic: an overview of systematic reviews. BMC Infect Dis.

[22] Li Y, Cao L, Zhang Z (2021). Reporting and methodological quality of COVID-19 systematic reviews needs to be improved: an evidence mapping. J Clin Epidemiol.

[23] Chen Y, Li L, Zhang Q (2021). Epidemiology, methodological quality, and reporting characteristics of systematic reviews and meta-analyses on coronavirus disease 2019: a cross-sectional study. Medicine.

[24] Cuschieri S (2019). The STROBE guidelines. Saudi J Anaesth.

[25] Iannizzi C, Dorando E, Burns J (2021). Methodological challenges for living systematic reviews conducted during the COVID-19 pandemic: a concept paper. J Clin Epidemiol.

[26] Lorenz RC, Matthias K, Pieper D (2019). A psychometric study found AMSTAR 2 to be a valid and moderately reliable appraisal tool. J Clin Epidemiol.

[27] Perry R, Whitmarsh A, Leach V, Davies P (2021). A comparison of two assessment tools used in overviews of systematic reviews: ROBIS versus AMSTAR-2. Syst Rev.

[28] Page MJ, McKenzie JE, Bossuyt PM (2021). The PRISMA 2020 statement: an updated guideline for reporting systematic reviews. BMJ (Clinical Research Ed).

[29] Zhu Y, Fan L, Zhang H (2016). Is the best evidence good enough: quality assessment and factor analysis of meta-analyses on depression. PLoS ONE.

[30] Akinwande O, Dikko HG, Agboola S (2015). Variance inflation factor: as a condition for the inclusion of suppressor variable(s) in regression analysis. Open J Stat.

[31] Allotey J, Chatterjee S, Kew T (2022). SARS-CoV-2 positivity in offspring and timing of mother-to-child transmission: living systematic review and meta-analysis. BMJ.

[32] Allotey J, Stallings E, Bonet M (2020). Clinical manifestations, risk factors, and maternal and perinatal outcomes of coronavirus disease 2019 in pregnancy: living systematic review and meta-analysis. BMJ.

[33] Amer YS, Titi MA, Godah MW (2022). International alliance and AGREE-ment of 71 clinical practice guidelines on the management of critical care patients with COVID-19: a living systematic review. J Clin Epidemiol.

[34] Amorim Dos Santos J, Normando AGC, da Carvalho Silva RL (2021). Oral manifestations in patients with covid-19: a 6-month update. J Dent Res..

[35] Ansems K, Grundeis F, Dahms K (2021). Remdesivir for the treatment of COVID-19. Cochrane Database Syst Rev.

[36] Asiimwe IG, Pushpakom S, Turner RM, Kolamunnage-Dona R, Jorgensen AL, Pirmohamed M (2021). Cardiovascular drugs and COVID-19 clinical outcomes: A living systematic review and meta-analysis. Br J Clin Pharmacol.

[37] Baladia E, Pizarro AB, Ortiz-Muñoz L, Rada G (2020). Vitamin C for COVID-19: a living systematic review. Medwave.

[38] Bartoszko JJ, Siemieniuk RAC, Kum E (2021). Prophylaxis against covid-19: living systematic review and network meta-analysis. BMJ.

[39] Bell V, Wade D (2021). Mental health of clinical staff working in high-risk epidemic and pandemic health emergencies a rapid review of the evidence and living meta-analysis. Soc Psychiatry Psychiatr Epidemiol.

[40] Bonardi O, Wang Y, Li K (2022). Effects of COVID-19 mental health interventions among children, adolescents, and adults not quarantined or undergoing treatment due to COVID-19 infection: a systematic review of randomised controlled trials. Can J Psychiatry.

[41] Brümmer LE, Katzenschlager S, Gaeddert M (2021). Accuracy of novel antigen rapid diagnostics for SARS-CoV-2: a living systematic review and meta-analysis. PLoS Med.

[42] Buitrago-Garcia D, Egli-Gany D, Counotte MJ (2020). Occurrence and transmission potential of asymptomatic and presymptomatic SARS-CoV-2 infections: a living systematic review and meta-analysis. PLoS Med.

[43] Bwire GM, Njiro BJ, Mwakawanga DL, Sabas D, Sunguya BF (2021). Possible vertical transmission and antibodies against SARS-CoV-2 among infants born to mothers with COVID-19: A living systematic review. J Med Virol.

[44] Cares-Marambio K, Montenegro-Jiménez Y, Torres-Castro R (2021). Prevalence of potential respiratory symptoms in survivors of hospital admission after coronavirus disease 2019 (COVID-19): A systematic review and meta-analysis. Chron Respir Dis.

[45] Centeno-Tablante E, Medina-Rivera M, Finkelstein JL (2021). Transmission of SARS-CoV-2 through breast milk and breastfeeding: a living systematic review. Ann N Y Acad Sci.

[46] Ceravolo MG, Andrenelli E, Arienti C (2021). Rehabilitation and COVID-19: rapid living systematic review by cochrane rehabilitation Field - third edition. Update as of June 30th, 2021. Eur J Phys Rehabil Med.

[47] Davidson M, Menon S, Chaimani A (2022). Interleukin-1 blocking agents for treating COVID-19. Cochrane Database Syst Rev.

[48] Deeks JJ, Dinnes J, Takwoingi Y (2020). Antibody tests for identification of current and past infection with SARS-CoV-2. Cochrane Database Syst Rev.

[49] Dong F, Liu HL, Dai N, Yang M, Liu JP (2021). A living systematic review of the psychological problems in people suffering from COVID-19. J Affect Disord.

[50] Dzinamarira T, Nkambule SJ, Hlongwa M (2022). Risk factors for COVID-19 infection among healthcare workers. A first report from a living systematic review and meta-analysis. Saf Health Work.

[51] Elvidge J, Summerfield A, Nicholls D, Dawoud D (2022). Diagnostics and treatments of COVID-19: a living systematic review of economic evaluations. Value Health.

[52] Ghosn L, Chaimani A, Evrenoglou T (2021). Interleukin-6 blocking agents for treating COVID-19: a living systematic review. Cochrane Database Syst Rev.

[53] Gómez-Ochoa SA, Franco OH, Rojas LZ (2021). COVID-19 in health-care workers: a living systematic review and meta-analysis of prevalence, risk factors, clinical characteristics, and outcomes. Am J Epidemiol.

[54] Griesel M, Wagner C, Mikolajewska A (2022). Inhaled corticosteroids for the treatment of COVID-19. Cochrane Database Syst Rev.

[55] Harder T, Koch J, Vygen-Bonnet S (2021). Efficacy and effectiveness of COVID-19 vaccines against SARS-CoV-2 infection: interim results of a living systematic review, 1 January to 14 May 2021. Euro Surveill.

[56] Harder T, Külper-Schiek W, Reda S (2021). Effectiveness of COVID-19 vaccines against SARS-CoV-2 infection with the Delta (B.1.617.2) variant: second interim results of a living systematic review and meta-analysis, 1 January to 25 August 2021. Euro Surveill.

[57] Helfand M, Fiordalisi C, Wiedrick J (2022). Risk for reinfection after SARS-CoV-2: a living, rapid review for american college of physicians practice points on the role of the antibody response in conferring immunity following SARS-CoV-2 infection. Ann Intern Med.

[58] Hernandez AV, Roman YM, Pasupuleti V, Barboza JJ, White CM (2020). Hydroxychloroquine or chloroquine for treatment or prophylaxis of COVID-19: a living systematic review. Ann Intern Med.

[59] Hussain S, Riad A, Singh A (2021). Global prevalence of COVID-19-associated mucormycosis (CAM): living systematic review and meta-analysis. J Fungi (Basel, Switzerland)..

[60] John A, Eyles E, Webb RT (2020). The impact of the COVID-19 pandemic on self-harm and suicidal behaviour: update of living systematic review. F1000Res.

[61] Juul S, Nielsen EE, Feinberg J (2020). Interventions for treatment of COVID-19: A living systematic review with meta-analyses and trial sequential analyses (The LIVING Project). PLoS Med.

[62] Kirkham AM, Monaghan M, Bailey AJM (2022). Mesenchymal stem/stromal cell-based therapies for COVID-19: First iteration of a living systematic review and meta-analysis: MSCs and COVID-19. Cytotherapy..

[63] Korang SK, von Rohden E, Veroniki AA (2022). Vaccines to prevent COVID-19: a living systematic review with trial sequential analysis and network meta-analysis of randomized clinical trials. PLoS ONE.

[64] Kreuzberger N, Hirsch C, Chai KL (2021). SARS-CoV-2-neutralising monoclonal antibodies for treatment of COVID-19. Cochrane Database Syst Rev.

[65] Langford BJ, So M, Raybardhan S (2020). Bacterial co-infection and secondary infection in patients with COVID-19: a living rapid review and meta-analysis. Clin Microbiol Infect.

[66] Mackey K, King VJ, Gurley S (2020). Risks and impact of angiotensin-converting enzyme inhibitors or angiotensin-receptor blockers on SARS-CoV-2 infection in adults: a living systematic review. Ann Intern Med.

[67] Maguire BJ, McLean ARD, Rashan S (2020). Baseline results of a living systematic review for COVID-19 clinical trial registrations. Wellcome Open Res.

[68] Melo AKG, Milby KM, Caparroz A (2021). Biomarkers of cytokine storm as red flags for severe and fatal COVID-19 cases: A living systematic review and meta-analysis. PLoS ONE.

[69] Michelen M, Manoharan L, Elkheir N (2021). Characterising long COVID: a living systematic review. BMJ Glob Health.

[70] Mikolajewska A, Fischer AL, Piechotta V (2021). Colchicine for the treatment of COVID-19. Cochrane Database Syst Rev.

[71] O'Byrne L, Webster KE, MacKeith S, Philpott C, Hopkins C, Burton MJ (2021). Interventions for the treatment of persistent post-COVID-19 olfactory dysfunction. Cochrane Database Syst Rev.

[72] Qiu X, Nergiz AI, Maraolo AE, Bogoch II, Low N, Cevik M (2021). The role of asymptomatic and pre-symptomatic infection in SARS-CoV-2 transmission-a living systematic review. Clin Microbiol Infect.

[73] Rada G, Corbalán J, Rojas P (2020). Cell-based therapies for COVID-19: a living, systematic review. Medwave.

[74] Rocha APD, Atallah ÁN, Pinto A (2020). COVID-19 and patients with immune-mediated inflammatory diseases undergoing pharmacological treatments: a rapid living systematic review. Sao Paulo Med J.

[75] Salameh JP, Leeflang MM, Hooft L (2020). Thoracic imaging tests for the diagnosis of COVID-19. Cochrane Database Syst Rev.

[76] Schlesinger S, Neuenschwander M, Lang A (2021). Risk phenotypes of diabetes and association with COVID-19 severity and death: a living systematic review and meta-analysis. Diabetologia.

[77] Schünemann HJ, Khabsa J, Solo K (2020). Ventilation techniques and risk for transmission of coronavirus disease, including COVID-19: a living systematic review of multiple streams of evidence. Ann Intern Med.

[78] Siemieniuk RA, Bartoszko JJ, Díaz Martinez JP (2021). Antibody and cellular therapies for treatment of covid-19: a living systematic review and network meta-analysis. BMJ.

[79] Siemieniuk RA, Bartoszko JJ, Ge L (2020). Drug treatments for covid-19: living systematic review and network meta-analysis. BMJ.

[80] Silveira FM, Mello ALR, da Silva FL (2022). Morphological and tissue-based molecular characterization of oral lesions in patients with COVID-19: a living systematic review. Arch Oral Biol.

[81] Soto-Cámara R, García-Santa-Basilia N, Onrubia-Baticón H (2021). Psychological impact of the COVID-19 pandemic on out-of-hospital health professionals: a living systematic review. J Clin Med.

[82] Stroehlein JK, Wallqvist J, Iannizzi C (2021). Vitamin D supplementation for the treatment of COVID-19: a living systematic review. Cochrane Database Syst Rev.

[83] Tleyjeh IM, Kashour Z, Riaz M, Hassett L, Veiga VC, Kashour T (2021). Efficacy and safety of tocilizumab in COVID-19 patients: a living systematic review and meta-analysis, first update. Clin Microbiol Infect.

[84] Valk SJ, Piechotta V, Chai KL (2020). Convalescent plasma or hyperimmune immunoglobulin for people with COVID-19: a rapid review. Cochrane Database Syst Rev.

[85] Verdejo C, Vergara-Merino L, Meza N (2020). Macrolides for the treatment of COVID-19: a living, systematic review. Medwave.

[86] Verdugo-Paiva F, Acuña MP, Solá I, Rada G (2020). Remdesivir for the treatment of COVID-19: a living systematic review. Medwave.

[87] Verdugo-Paiva F, Izcovich A, Ragusa M, Rada G (2020). Lopinavir-ritonavir for COVID-19: a living systematic review. Medwave.

[88] Wagner C, Griesel M, Mikolajewska A (2021). Systemic corticosteroids for the treatment of COVID-19. Cochrane Database Syst Rev.

[89] Webster KE, O'Byrne L, MacKeith S, Philpott C, Hopkins C, Burton MJ (2021). Interventions for the prevention of persistent post-COVID-19 olfactory dysfunction. Cochrane Database Syst Rev.

[90] Wilt TJ, Kaka AS, MacDonald R, Greer N, Obley A, Duan-Porter W (2021). Remdesivir for adults with COVID-19: a living systematic review for American college of physicians practice points. Ann Intern Med.

[91] Wynants L, Van Calster B, Collins GS (2020). Prediction models for diagnosis and prognosis of covid-19: systematic review and critical appraisal. BMJ.

[92] Xu W, Li X, Dong Y (2021). SARS-CoV-2 transmission in schools: an updated living systematic review (version 2; November 2020). J Glob Health.

[93] Yang J, D'Souza R, Kharrat A (2022). Coronavirus disease 2019 pandemic and pregnancy and neonatal outcomes in general population: a living systematic review and meta-analysis (updated Aug 14, 2021). Acta Obstet Gynecol Scand.

[94] Zhang X, Shang L, Fan G (2021). The efficacy and safety of Janus Kinase inhibitors for patients with COVID-19: a living systematic review and meta-analysis. Front Med.

[95] Zheng Q, Xu J, Gao Y (2022). Past, present and future of living systematic review: a bibliometrics analysis. BMJ Glob Health.

[96] Harvey LA, Dijkers MP (2019). Should trials that are highly vulnerable to bias be excluded from systematic reviews?. Spinal Cord.

[97] Murad MH, Wang Z, Chu H, Lin L, El Mikati IK, Khabsa J, Akl EA, Nieuwlaat R, Schuenemann HJ, Riaz IB. Proposed triggers for retiring a living systematic review. BMJ Evid Based Med. 2023:bmjebm-2022-112100. 10.1136/bmjebm-2022-112100 PMC10579491 36889900

[98] Kousha K, Thelwall M, Bickley M (2022). The high scholarly value of grey literature before and during Covid-19. Scientometrics.

[99] Cochrane collaboration[homepage on the Internet]. Guidance for the production and publication of Cochrane living systematic reviews: Cochrane Reviews in living mode. 2019; Available from:https://community.cochrane.org/sites/default/files/uploads/inline-files/Transform/201912_LSR_Revised_Guidance.pdf. Accessed 03–09, 2022.

[100] Helliwell JA, Thompson J, Smart N, Jayne DG, Chapman SJ (2022). Duplication and nonregistration of COVID-19 systematic reviews: bibliometric review. Health Sci Rep.

[101] Sideri S, Papageorgiou SN, Eliades T (2018). Registration in the international prospective register of systematic reviews (PROSPERO) of systematic review protocols was associated with increased review quality. J Clin Epidemiol.

[102] Ge L, Tian JH, Li YN (2018). Association between prospective registration and overall reporting and methodological quality of systematic reviews: a meta-epidemiological study. J Clin Epidemiol.

[103] Akl EA, El Khoury R, Khamis AM (2023). The life and death of living systematic reviews: a methodological survey. J Clin Epidemiol.

