# Supplementary material for: Methodological quality and reporting quality of COVID-19 living systematic review: a cross-sectional study
Source: BMC Med Res Methodol. 2023 Jul 31;23:175. doi: 10.1186/s12874-023-01980-y (PMC10388517; doi:10.1186/s12874-023-01980-y)
Supplement: Supplementary file 1 — Additional file 1: Appendix I. STROBE Checklists. Appendix II. SEARCH STRATEGIES. Appendix III. PRISMA statement 2020 assessment results. Appendix IV. AMSTAR-2 assessment results. Appendix V. Characteristics of the included studies. Appendix VI. List of the excluded studies. [file 12874_2023_1980_MOESM1_ESM.pdf]

Appendix I：STROBE Checklists 2
Appendix II：SEARCH STRATEGIES 4
Appendix III：PRISMA statement 2020 assessment results 7
Appendix IV：AMSTAR-2 assessment results 12
Appendix V：Characteristics of the included studies 17
Appendix VI：List of the excluded studies 22

Appendix I：STROBE Checklists

Section and Topic	Item	Recommendation	Location
**Title and abstract**	1	(a) Indicate the study's design with a commonly used term in the title or the abstract	Line 1-2
		(b) Provide in the abstract an informative and balanced summary of what was done and what was found	Line 25-55
**Introduction**			
Background/rationale	2	Explain the scientific background and rationale for the investigation being reported	Line 73-155
Objectives	3	State specific objectives, including any prespecified hypotheses	Line 116-120
**Methods**			
Study design	4	Present key elements of study design early in the paper	Line 122
Setting	5	Describe the setting, locations, and relevant dates, including periods of recruitment, exposure, follow-up, and data collection	Line 129-140
Participants	6	(a) Give the eligibility criteria, and the sources and methods of selection of participants	Line 141-147
Variables	7	Clearly define all outcomes, exposures, predictors, potential confounders, and effect modifiers. Give diagnostic criteria, if applicable	Line 157-169
Data sources/ measurement	8*	For each variable of interest, give sources of data and details of methods of assessment (measurement). Describe comparability of assessment methods if there is more than one group	Line 170-207
Bias	9	Describe any efforts to address potential sources of bias	Line 149-156
Study size	10	Explain how the study size was arrived at	Line 136
Quantitative variables	11	Explain how quantitative variables were handled in the analyses. If applicable, describe which groupings were chosen and why	Line 209-218
Statistical methods	12	(a) Describe all statistical methods, including those used to control for confounding	Line 209-218
		(b) Describe any methods used to examine subgroups and interactions	/
		(c) Explain how missing data were addressed	Line 136-139
		(d) If applicable, describe analytical methods taking account of sampling strategy	/
		(e) Describe any sensitivity analyses	Line 219-228
**Results**			
Participants	13*	(a) Report numbers of individuals at each stage of study—eg numbers potentially eligible, examined for eligibility, confirmed eligible, included in the study, completing follow-up, and analysed	Line 231-238
		(b) Give reasons for non-participation at each stage	Line 237-238
		(c) Consider use of a flow diagram	Figure 1
Descriptive data	14*	(a) Give characteristics of study participants (eg demographic, clinical, social) and information on exposures and potential confounders	Line 240-249

		(b) Indicate number of participants with missing data for each variable of interest	/
Outcome data	15*	Report numbers of outcome events or summary measures	Figure 3 and Figure 5
Main results	16	(a) Give unadjusted estimates and, if applicable, confounder-adjusted estimates and their precision (eg, 95% confidence interval). Make clear which confounders were adjusted for and why they were included	Line 251-256, Line 265-269
		(b) Report category boundaries when continuous variables were categorized	Line 253-256
		(c) If relevant, consider translating estimates of relative risk into absolute risk for a meaningful time period	/
Other analyses	17	Report other analyses done—eg analyses of subgroups and interactions, and sensitivity analyses	Line 257-263, Line 270-280, Line 285-290
**Discussion**			
Key results	18	Summarise key results with reference to study objectives	Line 293-301
Limitations	19	Discuss limitations of the study, taking into account sources of potential bias or imprecision. Discuss both direction and magnitude of any potential bias	Line 409-422
Interpretation	20	Give a cautious overall interpretation of results considering objectives, limitations, multiplicity of analyses, results from similar studies, and other relevant evidence	Line 302-406
Generalisability	21	Discuss the generalisability (external validity) of the study results	Line 403-406
**Other information**			
Funding	22	Give the source of funding and the role of the funders for the present study and, if applicable, for the original study on which the present article is based	Line 446-448

Appendix II：SEARCH STRATEGIES

	Medline Search Strategy	result
1	(contin* adj5 (updat* or search*)).af.	5385
2	(meta-analysis or review).pt,tw.	3323560
3	(living adj5 (evidence or SR or review* or overview* or over-view* or metaanaly* or meta analy* or metanaly* or meta-analy*)).af.	2958
4	LSR.af.	925
5	1 and 2	1740
6	3 or 4 or 5	5565
7	exp Coronavirus Infections/	167199
8	exp coronavirinae/	136891
9	(coronavirus* or corona virus* or OC43 or NL63 or 229E or HKU1 or HCoV* or ncov* or covid* or sars-cov* or sarscov* or Sars-coronavirus* or Severe Acute Respiratory Syndrome Coronavirus*).mp.	176447
10	7 or 8 or 9	181949
11	10 not (SARS or SARS-CoV or MERS or MERS-CoV or Middle East respiratory syndrome or camel* or dromedar* or equine or coronary or coronal or covidence* or covidien or 2 influenza virus or HIV or bovine or calves or TGEV or feline or porcine or BCoV or PED or PEDV or PDCoV or FIPV or FCoV or SADS-CoV or canine or CCov or zoonotic or avian influenza or H1N1 or H5N1 or H5N6 or IBV or murine corona*).mp.	36060
12	((pneumonia or covid* or coronavirus* or corona virus* or ncov* or 2019-ncov or sars*).mp. or exp pneumonia/) and Wuhan.mp.	4434
13	(2019-ncov or ncov19 or ncov-19 or 2019-novel CoV or sars-cov2 or sars-cov-2 or sarscov2 or sarscov-2 or Sars-coronavirus2 or Sars-coronavirus-2 or SARS-like coronavirus* or coronavirus-19 or covid19 or covid-19 or covid 2019 or ((novel or new or nouveau) adj2 (CoV on nCoV or covid or coronavirus* or corona virus or Pandemi*2)) or ((covid or covid19 or covid-19) and pandemic*2) or (coronavirus* and pneumonia)).mp.	163473
14	12 or 13	163496
15	11 or 14	169700
16	6 and 15	254

	Embase Search Strategy	result
1	exp systematic review/	343464
2	systematic review.af.	245788
3	(living adj5 (evidence or SR or review* or overview* or over-view* or metaanaly* or meta analy* or metanaly* or meta-analy*)).af.	2958
4	(metaanaly* or meta analy* or metanaly* or meta-analy*).af.	385407
5	(contin* adj5 (updat* or search*)).af.	8992
6	LSR.af.	2568
7	(living adj5 ('evidence' or 'sr' or 'review*' or 'overview*' or 'over view*' or 'metanaly*' or 'metaanaly*' or 'meta analy*')).af.	4283
8	1 or 2 or 3 or 4	612644
9	5 or 8	576
10	6 or 7 or 9	7364
11	exp Coronavirus Infections/	232527
12	exp coronavirinae/	85528
13	(coronavirus* or corona virus* or OC43 or NL63 or 229E or HKU1 or HCoV* or ncov* or covid* or sars-cov* or sarscov* or Sars-coronavirus* or Severe Acute Respiratory Syndrome Coronavirus*).mp.	308327
14	11 or 12 or 13	316072
15	14 not (SARS or SARS-CoV or MERS or MERS-CoV or Middle East respiratory syndrome or camel* or dromedar* or equine or coronary or coronal or covidence* or covidien or 2 influenza virus or HIV or bovine or calves or TGEV or feline or porcine or BCoV or PED or PEDV or PDCoV or FIPV or FCoV or SADS-CoV or canine or CCov or zoonotic or avian influenza or H1N1 or H5N1 or H5N6 or IBV or murine corona*).mp.	180417
16	((pneumonia or covid* or coronavirus* or corona virus* or ncov* or 2019-ncov or sars*).mp. or exp pneumonia/) and Wuhan.mp.	7325
17	(2019-ncov or ncov19 or ncov-19 or 2019-novel CoV or sars-cov2 or sars-cov-2 or sarscov2 or sarscov-2 or Sars-coronavirus2 or Sars-coronavirus-2 or SARS-like coronavirus* or coronavirus-19 or covid19 or covid-19 or covid 2019 or ((novel or new or nouveau) adj2 (CoV on nCoV or covid or coronavirus* or corona virus or Pandemi*2)) or ((covid or covid19 or covid-19) and pandemic*2) or (coronavirus* and pneumonia)).mp.	262939
18	16 or 17	263056

19	15 or 18	291308
20	10 and 19	329

	**Cochrane library Search Strategy**	**result**
1	(contin* adj5 (updat* or search*)).af.	343
2	LSR.af	18
3	(living adj5 (evidence or sr or review* or overview* or "over-view*" or metanaly* or metaanaly* or "meta-analy*")).af.	250
4	"coronavirus*" or "coronovirus*" or "coronavirinae*" or "2019-nCoV" or 2019nCoV or nCoV2019 or COVID19 or SARSCov19 or SARSCoV2	103
5	1 or 2 or 3	559
6	4 and 5	39

	**CNKI**	**VIP**	**Wanfang**
**Strategy**	SU=动态系统评价 OR SU=动态系统综述 OR SU=动态 meta 分析 OR SU=动态荟萃分析 OR SU=实时系统评价 OR SU=实时系统综述 OR SU=实时 meta 分析 OR SU=实时荟萃分析	M=动态系统评价 OR M=动态系统综述 OR M=动态 meta 分析 OR M=动态荟萃分析 OR M=实时系统评价 OR M=实时系统综述 OR M=实时 meta 分析 OR M=实时荟萃分析	主题:(动态 Meta 分析) + 主题:(动态系统评价) + 主题:(动态系统综述) + 主题:(动态荟萃分析) + 主题:(实时 Meta 分析) + 主题:(实时系统评价) + 主题:(实时系统综述) + 主题:(实时荟萃分析)
**Result**	20	480	10

Appendix III：PRISMA statement 2020 assessment results

Study	Items 1	Items 2	Items 3	Items 4	Items 5	Items 6	Items 7	Items 8	Items 9	Items 10	Items 11	Items 12	Items 13	Items 14	Items 15	Items 16	Items 17	Items 18	Items 19	Items 20	Items 21	Items 22	Items 23	Items 24	Items 25	Items 26	Items 27
Wynants 2020	Y	Y	Y	Y	Y	Y	PY	PY	Y	Y	Y	N	N	N	N	PY	Y	PY	N	N	N	N	Y	Y	Y	Y	Y
Allotey 2020	Y	Y	Y	Y	Y	Y	Y	Y	Y	PY	Y	PY	Y	N	N	PY	Y	Y	Y	Y	N	N	Y	Y	Y	Y	Y
Allotey 2022	Y	Y	Y	Y	Y	Y	PY	Y	Y	Y	PY	Y	Y	N	N	PY	Y	Y	Y	Y	N	N	Y	Y	Y	Y	Y
Wilt 2020	Y	Y	Y	Y	Y	Y	Y	PY	Y	PY	Y	PY	PY	N	Y	PY	Y	Y	Y	Y	N	Y	Y	N	Y	N	N
Schünemann 2020	Y	Y	Y	Y	Y	Y	Y	Y	Y	Y	Y	N	PY	N	Y	PY	Y	Y	Y	Y	N	Y	Y	Y	Y	Y	N
Mackey 2020	Y	Y	Y	Y	Y	Y	Y	PY	Y	Y	Y	Y	PY	N	N	PY	PY	Y	Y	Y	Y	N	Y	PY	Y	Y	N
Hernandez 2020	Y	Y	PY	Y	Y	Y	PY	Y	Y	Y	Y	Y	N	N	Y	PY	Y	Y	Y	Y	N	Y	Y	N	Y	Y	N
Helfand 2022	N	Y	Y	Y	PY	Y	Y	PY	PY	Y	N	Y	PY	Y	Y	PY	Y	Y	Y	Y	Y	Y	Y	Y	Y	N	N
Buitrago-Garcia 2020	Y	Y	Y	Y	Y	Y	Y	Y	Y	Y	Y	Y	Y	N	N	PY	Y	Y	Y	Y	N	N	Y	Y	Y	Y	Y
Schlesinger 2021	Y	Y	Y	Y	Y	Y	PY	Y	Y	Y	Y	Y	Y	Y	Y	Y	Y	Y	Y	Y	Y	Y	Y	Y	Y	N	Y
Griesel 2022	Y	Y	Y	Y	Y	Y	Y	Y	Y	Y	Y	Y	Y	Y	Y	Y	Y	Y	Y	Y	Y	Y	Y	Y	Y	Y	Y

Webster 2021	Y	Y	Y	Y	Y	Y	Y	Y	Y	Y	Y	Y	Y	Y	Y	Y	Y	Y	Y	Y	Y	Y	Y	Y	Y	Y	Y
Wagner 2021	Y	Y	Y	Y	Y	Y	Y	Y	Y	Y	Y	Y	Y	Y	Y	Y	Y	Y	Y	Y	Y	Y	Y	Y	Y	Y	Y
Valk 2020	Y	Y	Y	Y	Y	Y	Y	Y	Y	Y	Y	Y	Y	Y	Y	Y	Y	Y	Y	Y	Y	Y	Y	Y	Y	Y	Y
Stroehlein 2021	Y	Y	Y	Y	Y	Y	Y	Y	Y	Y	Y	Y	Y	Y	Y	Y	Y	Y	Y	Y	Y	Y	Y	Y	Y	Y	Y
O'Byrne 2021	Y	Y	Y	Y	Y	Y	Y	Y	Y	Y	Y	Y	Y	Y	Y	Y	Y	Y	Y	Y	Y	Y	Y	Y	Y	Y	Y
Mikolajewska 2021	Y	Y	Y	Y	Y	Y	Y	Y	Y	Y	Y	Y	Y	Y	Y	Y	Y	Y	Y	Y	Y	Y	Y	Y	Y	Y	Y
Kreuzberger 2021	Y	Y	Y	Y	Y	Y	Y	Y	Y	Y	Y	Y	Y	Y	Y	Y	Y	Y	Y	Y	Y	Y	Y	Y	Y	Y	Y
Ghosn 2021	Y	Y	Y	Y	Y	Y	Y	Y	Y	Y	Y	Y	Y	Y	Y	Y	Y	Y	Y	Y	Y	Y	Y	Y	Y	Y	Y
Davidson 2022	Y	Y	Y	Y	Y	Y	Y	Y	Y	Y	Y	Y	Y	Y	Y	Y	Y	Y	Y	Y	Y	Y	Y	Y	Y	Y	Y
Ansems 2021	Y	Y	Y	Y	Y	Y	Y	Y	Y	Y	Y	Y	Y	Y	Y	Y	Y	Y	Y	Y	Y	Y	Y	Y	Y	Y	Y
Tleyjeh 2021	Y	PY	Y	Y	Y	Y	Y	Y	Y	PY	Y	Y	Y	Y	Y	PY	Y	Y	Y	Y	Y	Y	PY	N	Y	Y	N
Qiu 2021	Y	Y	Y	Y	Y	Y	PY	Y	Y	PY	Y	Y	Y	N	N	PY	Y	Y	Y	Y	N	N	Y	Y	Y	Y	N
Langford 2020	Y	PY	Y	Y	Y	Y	PY	Y	Y	Y	N	PY	Y	N	N	PY	Y	N	Y	Y	N	N	Y	Y	Y	Y	N
Amer 2021	Y	PY	Y	Y	PY	Y	Y	Y	PY	Y	Y	Y	Y	N	N	PY	Y	Y	PY	Y	N	N	Y	Y	Y	Y	N
Harder 2021	Y	N	Y	Y	Y	Y	Y	Y	PY	Y	PY	N	N	N	N	PY	Y	Y	Y	Y	N	N	PY	Y	N	Y	N
Harder 2021	Y	N	Y	Y	Y	Y	Y	Y	PY	Y	PY	Y	Y	Y	N	PY	Y	Y	Y	Y	Y	N	PY	Y	N	Y	N

Santos 2020	Y	Y	Y	Y	Y	Y	Y	Y	Y	Y	Y	Y	Y	N	Y	Y	Y	Y	Y	Y	N	Y	Y	Y	Y	Y	N
Hussain 2021	Y	PY	Y	Y	Y	Y	Y	Y	Y	Y	PY	PY	Y	Y	Y	Y	Y	Y	Y	Y	Y	PY	Y	N	Y	Y	Y
Elvidge 2022	Y	PY	Y	Y	Y	Y	Y	Y	PY	PY	PY	N	N	N	N	Y	Y	Y	Y	Y	N	N	Y	Y	Y	Y	N
Centeno-Tablante 2020	Y	PY	Y	Y	Y	Y	Y	Y	Y	Y	N	PY	PY	N	Y	Y	Y	N	Y	Y	N	PY	Y	Y	Y	Y	N
Michelen 2021	Y	Y	Y	Y	Y	Y	Y	Y	Y	Y	PY	Y	Y	Y	N	PY	Y	Y	Y	Y	Y	N	Y	Y	Y	Y	Y
Kirkham 2022	Y	PY	Y	Y	Y	Y	Y	Y	Y	Y	PY	Y	Y	PY	N	PY	Y	Y	Y	Y	PY	N	Y	Y	Y	Y	Y
Zhang 2022	Y	Y	Y	Y	Y	Y	Y	Y	Y	Y	PY	Y	Y	Y	N	PY	Y	Y	Y	Y	Y	N	Y	Y	Y	Y	Y
Gómez-Ochoa 2021	Y	PY	Y	Y	Y	Y	Y	Y	Y	Y	Y	Y	Y	N	N	PY	Y	Y	Y	Y	N	N	Y	N	Y	Y	N
Dong 2021	Y	PY	Y	Y	Y	Y	Y	Y	Y	Y	Y	Y	Y	Y	N	PY	Y	Y	Y	Y	Y	Y	Y	PY	Y	Y	N
Xu 2020	Y	PY	Y	Y	Y	Y	Y	PY	Y	PY	PY	PY	Y	Y	N	PY	Y	Y	Y	Y	Y	N	Y	Y	Y	Y	N
Bonardi 2022	Y	PY	Y	Y	Y	Y	Y	Y	Y	Y	PY	PY	N	N	N	Y	Y	Y	Y	Y	N	N	Y	Y	Y	Y	Y
Asiimwe 2021	Y	PY	Y	Y	Y	Y	N	Y	Y	Y	Y	Y	Y	Y	Y	PY	Y	PY	Y	Y	Y	PY	Y	Y	Y	Y	Y
Bell 2020	Y	PY	Y	Y	PY	PY	N	Y	Y	PY	Y	PY	Y	Y	N	PY	Y	Y	Y	Y	Y	N	Y	N	N	Y	Y
Soto-Cámara 2021	Y	PY	Y	Y	Y	Y	Y	Y	Y	PY	Y	N	PY	N	N	Y	Y	Y	PY	Y	N	N	Y	PY	Y	Y	Y

Yang 2021	Y	PY	Y	Y	Y	Y	Y	Y	Y	Y	PY	Y	Y	Y	N	Y	Y	Y	Y	Y	Y	N	Y	Y	N	Y	N
Melo 2021	Y	Y	Y	Y	Y	Y	Y	Y	Y	Y	Y	Y	Y	Y	N	Y	Y	Y	Y	Y	Y	N	Y	Y	Y	Y	Y
Ceravolo 2020	Y	PY	Y	PY	Y	Y	Y	Y	Y	Y	Y	PY	PY	N	Y	PY	Y	N	PY	Y	N	Y	Y	Y	N	Y	N
Dzinamarira 2022	Y	PY	Y	Y	Y	Y	PY	Y	Y	Y	Y	Y	Y	Y	Y	PY	Y	Y	Y	PY	N	N	Y	Y	N	Y	N
Silveira 2022	Y	PY	Y	Y	Y	Y	PY	Y	PY	Y	PY	N	N	N	N	PY	Y	Y	Y	Y	N	N	Y	Y	Y	Y	Y
Cares-Marambio 2021	Y	PY	Y	Y	Y	Y	N	Y	Y	Y	Y	PY	PY	N	N	PY	Y	Y	Y	Y	N	N	Y	Y	Y	Y	N
Bwire 2020	Y	PY	Y	Y	Y	Y	Y	Y	PY	PY	PY	Y	PY	PY	N	PY	Y	N	Y	PY	N	N	Y	Y	Y	Y	Y
Rocha 2020	Y	PY	Y	Y	Y	Y	Y	Y	Y	PY	Y	N	N	N	N	Y	Y	Y	Y	Y	N	PY	Y	Y	Y	N	N
Verdugo-Paiva 2020	Y	PY	Y	Y	Y	Y	Y	Y	Y	Y	Y	Y	Y	N	Y	Y	Y	Y	Y	Y	N	Y	Y	Y	Y	Y	Y
Verdugo-Paiva 2020	Y	Y	Y	Y	Y	Y	Y	Y	Y	Y	Y	Y	Y	N	Y	Y	Y	Y	Y	Y	N	Y	Y	Y	Y	Y	Y
Verdejo 2020	Y	Y	Y	Y	Y	Y	N	Y	Y	Y	Y	Y	Y	N	Y	Y	Y	Y	Y	Y	N	Y	Y	Y	Y	Y	Y
Rada 2020	Y	Y	Y	Y	Y	Y	Y	Y	Y	Y	Y	Y	Y	N	Y	Y	Y	N	N	N	N	N	PY	Y	Y	Y	Y
Maguire 2020	Y	Y	Y	Y	Y	Y	PY	Y	Y	N	PY	Y	PY	N	N	PY	PY	N	N	PY	N	N	Y	PY	Y	Y	Y
John 2020	Y	Y	Y	Y	Y	Y	PY	Y	Y	PY	PY	PY	PY	N	N	PY	Y	N	Y	Y	N	N	Y	Y	Y	Y	Y

Baladia 2020	Y	Y	Y	Y	Y	Y	Y	Y	N	PY	N	N	N	N	PY	Y	PY	N	N	N	N	N	Y	Y	Y	Y	Y
Siemieniuk (2020)	Y	Y	Y	Y	Y	Y	Y	Y	Y	Y	Y	Y	Y	PY	Y	Y	Y	Y	Y	Y	N	Y	Y	Y	Y	Y	Y
Siemieniuk (2021)	Y	Y	Y	Y	Y	Y	Y	Y	Y	Y	Y	Y	Y	PY	Y	Y	Y	Y	Y	Y	Y	Y	Y	Y	Y	Y	Y
Bartoszko	Y	Y	Y	Y	Y	Y	Y	Y	Y	Y	Y	Y	Y	PY	Y	Y	Y	Y	Y	Y	N	Y	Y	Y	Y	Y	Y
Juul	Y	Y	Y	PY	N	Y	Y	Y	Y	PY	Y	Y	Y	PY	Y	Y	Y	Y	Y	Y	N	Y	Y	Y	Y	Y	Y
Brümmer	Y	Y	Y	Y	Y	Y	Y	Y	Y	Y	PY	Y	Y	Y	N	Y	Y	Y	Y	Y	Y	N	Y	Y	Y	Y	Y
Salameh	Y	Y	Y	Y	Y	Y	Y	Y	Y	Y	Y	Y	Y	Y	N	Y	Y	Y	Y	Y	N	N	Y	Y	Y	Y	Y
Deeks	Y	Y	Y	Y	Y	Y	Y	Y	Y	Y	Y	Y	Y	N	Y	Y	Y	Y	Y	Y	PY	N	Y	Y	Y	Y	Y
Korang	Y	Y	Y	Y	Y	Y	Y	Y	Y	PY	PY	PY	Y	PY	Y	Y	Y	Y	Y	Y	N	N	Y	Y	Y	Y	Y

Note: Y=YES, N=NO, PY= PARTIALLY YES

Appendix IV：AMSTAR-2 assessment results

Author	Items 1	Items 2	Items 3	Items 4	Items 5	Items 6	Items 7	Items 8	Items 9	Items 10	Items 11	Items 12	Items 13	Items 14	Items 15	Items 16	Total
Wynants 2020	Yes	Partly Yes	Yes	Yes	No	Yes	No	Yes	Yes	No	No meta-analysis	No meta-analysis	Yes	No	No meta-analysis	Yes	Very low
Siemieniuk 2020	Yes	Yes	Yes	Yes	Yes	Yes	No	Partly Yes	Yes	No	Yes	No	No	No	No	Yes	Very low
Siemieniuk 2021	Yes	Partly Yes	Yes	Yes	Yes	Yes	Yes	Yes	Yes	No	Yes	Yes	Yes	No	No	Yes	Very low
Bartoszko 2021	Yes	Partly Yes	Yes	Yes	Yes	Yes	Yes	Yes	Yes	No	Yes	Yes	Yes	No	No	Yes	Very low
Allotey 2020	Yes	Yes	Yes	Yes	Yes	Yes	No	Yes	Yes	No	Yes	Yes	No	No	No	Yes	Very low
Allotey 2022	Yes	Partly Yes	No	Yes	Yes	Yes	No	Yes	Yes	No	Yes	Yes	Yes	Yes	No	Yes	Very low
Wilt 2020	Yes	No	No	Yes	No	Yes	No	Yes	Yes	No	No meta-analysis	No meta-analysis	No	No	No meta-analysis	Yes	Very low
Schünemann 2020	Yes	Partly Yes	Yes	Yes	Yes	Yes	No	Partly Yes	Yes	Yes	Yes	No	Yes	No	No	Yes	Very low

Mackey 2020	Yes	Partly Yes	No	Yes	Yes	Yes	No	Partly Yes	Yes	No	No meta-analysis	No meta-analysis	Yes	No	No meta-analysis	Yes	Very low
Hernandez 2020	Yes	Partly Yes	Yes	Yes	Yes	Yes	No	Yes	Yes	No	No meta-analysis	No meta-analysis	Yes	No	No meta-analysis	Yes	Very low
Helfand 2022	Yes	Yes	Yes	Yes	No	No	No	Yes	Yes	No	Yes	Yes	Yes	Yes	Yes	Yes	low
Juul 2020	Yes	Yes	Yes	Yes	Yes	Yes	Yes	Yes	Yes	No	Yes	Yes	Yes	Yes	Yes	Yes	High
Buitrago-Garcia 2020	Yes	Partly Yes	Yes	Yes	Yes	Yes	No	Yes	Yes	No	Yes	No	No	Yes	Yes	Yes	Very low
Brümmer 2021	Yes	Partly Yes	Yes	Yes	Yes	Yes	Yes	Yes	Yes	Yes	Yes	No	Yes	Yes	Yes	Yes	low
Schlesinger 2021	Yes	Yes	Yes	Yes	Yes	Yes	Yes	Yes	Yes	No	Yes	Yes	Yes	Yes	Yes	Yes	High
Griesel 2022	Yes	Yes	Yes	Yes	Yes	Yes	Yes	Yes	Yes	Yes	Yes	Yes	Yes	Yes	Yes	Yes	High
Webster 2021	Yes	Yes	Yes	Yes	Yes	Yes	Yes	Yes	Yes	Yes	No meta-analysis	No meta-analysis	Yes	Yes	No meta-analysis	Yes	High
Wagner 2021	Yes	Yes	Yes	Yes	Yes	Yes	Yes	Yes	Yes	Yes	Yes	Yes	Yes	Yes	Yes	Yes	High
Valk 2020	Yes	Yes	Yes	Yes	Yes	Yes	Yes	Yes	Yes	Yes	Yes	Yes	Yes	Yes	Yes	Yes	High
Stroehlein 2021	Yes	Yes	Yes	Yes	Yes	Yes	Yes	Yes	Yes	Yes	No meta-analysis	No meta-analysis	Yes	Yes	No meta-analysis	Yes	High

Salameh 2020	Yes	Yes	Yes	Yes	Yes	Yes	Yes	Yes	Yes	No	Yes	Yes	Yes	Yes	No	Yes	low
O'Byrne 2021	Yes	Yes	Yes	Yes	Yes	Yes	Yes	Yes	Yes	Yes	No meta-analysis	No meta-analysis	Yes	No	No meta-analysis	Yes	High
Mikolajewska 2021	Yes	Yes	Yes	Yes	Yes	Yes	Yes	Yes	Yes	Yes	No meta-analysis	No meta-analysis	Yes	Yes	No meta-analysis	Yes	High
Kreuzberger 2021	Yes	Yes	Yes	Yes	Yes	Yes	Yes	Yes	Yes	Yes	No meta-analysis	No meta-analysis	Yes	Yes	No meta-analysis	Yes	High
Ghosn 2021	Yes	Yes	Yes	Yes	Yes	Yes	Yes	Yes	Yes	Yes	Yes	Yes	Yes	Yes	Yes	Yes	High
Deeks 2020	Yes	Yes	Yes	Yes	Yes	Yes	Yes	Yes	Yes	Yes	Yes	No	No	Yes	Yes	Yes	low
Davidson 2022	Yes	Yes	Yes	Yes	Yes	Yes	Yes	Yes	Yes	Yes	Yes	Yes	Yes	No	Yes	Yes	High
Ansems 2021	Yes	Yes	Yes	Yes	Yes	Yes	Yes	Yes	Yes	Yes	Yes	Yes	Yes	Yes	Yes	Yes	High
Tleyjeh 2021	Yes	No	No	Yes	Yes	Yes	No	Yes	Yes	No	Yes	Yes	Yes	No	No	Yes	Very low
Qiu 2021	Yes	Partly Yes	Yes	Yes	Yes	Yes	No	Yes	Yes	No	Yes	No	Yes	No	No	Yes	Very low
Langford 2020	Yes	Partly Yes	Yes	Yes	Yes	Yes	No	Yes	No	No	Yes	No	No	No	No	Yes	Very low
Amer 2021	Yes	Partly Yes	Yes	Yes	Yes	Yes	No	Yes	Yes	No	No meta-analysis	No meta-analysis	Yes	No	No meta-analysis	Yes	Very low

Harder 2021	Yes	Yes	Yes	Yes	Yes	No	No	Yes	Yes	No	No meta-analysis	No meta-analysis	Yes	No	No meta-analysis	No	low
Harder 2021	Yes	Yes	Yes	Yes	Yes	No	No	Yes	Yes	No	Yes	No	Yes	Yes	Yes	No	low
Santos 2020	Yes	Yes	Yes	Yes	Yes	Yes	Yes	Yes	Yes	No	Yes	Yes	No	Yes	No	Yes	Very low
Hussain 2021	Yes	No	No	Yes	Yes	Yes	Yes	Yes	Yes	No	Yes	No	No	No	No	Yes	Very low
Elvidge 2022	Yes	Partly Yes	Yes	Yes	Yes	No	Yes	Yes	Yes	No	No meta-analysis	No meta-analysis	Yes	Yes	No meta-analysis	Yes	low
Centeno-Tablante 2020	Yes	Yes	Yes	Yes	Yes	Yes	Yes	Yes	No	No	No meta-analysis	No meta-analysis	No	No	No meta-analysis	Yes	Very low
Michelen 2021	Yes	Yes	Yes	Yes	Yes	Yes	No	Yes	Yes	Yes	Yes	Yes	Yes	Yes	Yes	Yes	low
Kirkham 2022	Yes	Partly Yes	No	Yes	Yes	Yes	No	Yes	Yes	No	Yes	No	Yes	Yes	Yes	Yes	Very low
Zhang 2022	Yes	Yes	Yes	Yes	Yes	Yes	No	Yes	Yes	No	Yes	No	Yes	No	Yes	Yes	low
Gómez-Ochoa 2021	Yes	No	No	Yes	Yes	Yes	No	Yes	Yes	No	Yes	No	Yes	Yes	No	Yes	Very low
Dong 2021	Yes	Partly Yes	No	Yes	Yes	Yes	No	Yes	Yes	No	Yes	No	No	Yes	Yes	Yes	Very low
Xu 2020	Yes	Partly Yes	No	Yes	Yes	Yes	No	Yes	Yes	No	Yes	No	Yes	Yes	Yes	Yes	Very low

Bonardi 2022	Yes	Yes	Yes	Yes	Yes	Yes	Yes	Yes	Yes	No	No meta-analysis	No meta-analysis	Yes	No	No meta-analysis	Yes	Moderate
Asiimwe 2021	Yes	Yes	Yes	Yes	Yes	Yes	No	Yes	Yes	No	Yes	No	Yes	Yes	Yes	Yes	low
Bell 2020	Yes	No	No	Yes	Yes	Yes	No	Yes	Yes	No	Yes	No	Yes	Yes	Yes	No	Very low
Soto-Cámara 2021	Yes	Partly Yes	Yes	Yes	Yes	Yes	No	Yes	Yes	No	No meta-analysis	No meta-analysis	Yes	Yes	No meta-analysis	Yes	Very low
Yang 2021	Yes	Partly Yes	Yes	Yes	Yes	Yes	Yes	Yes	Yes	No	Yes	No	No	Yes	Yes	No	Very low
Melo 2021	Yes	Yes	No	Yes	Yes	Yes	Yes	Yes	Yes	No	Yes	No	Yes	Yes	Yes	Yes	Moderate
Korang 2022	Yes	Yes	Yes	Yes	Yes	Yes	Yes	Yes	Yes	No	Yes	Yes	Yes	Yes	Yes	Yes	High
Ceravolo 2020	Yes	Yes	Yes	Yes	Yes	Yes	No	Yes	Yes	No	No meta-analysis	No meta-analysis	No	No	No meta-analysis	No	Very low
Dzinamarira 2022	Yes	Yes	Yes	Yes	Yes	Yes	No	Yes	Yes	No	Yes	No	Yes	Yes	Yes	No	low
Silveira 2022	Yes	Partly Yes	No	Yes	Yes	No	No	Yes	Yes	No	No meta-analysis	No meta-analysis	No	No	No meta-analysis	Yes	Very low
Cares-Marambio 2021	Yes	Partly Yes	Yes	Yes	Yes	Yes	No	Yes	Yes	No	Yes	No	No	Yes	No	Yes	Very low

Bwire 2020	Yes	Yes	Yes	Yes	Yes	No	No	Yes	No	No	No meta-analysis	No meta-analysis	Yes	No	No meta-analysis	Yes	Very low
Rocha 2020	Yes	Yes	Yes	Yes	Yes	Yes	Yes	Yes	Yes	No	No meta-analysis	No meta-analysis	Yes	No	No meta-analysis	Yes	Moderate
Verdugo-Paiva 2020	Yes	Yes	Yes	Yes	Yes	Yes	Yes	Yes	Yes	Yes	Yes	Yes	Yes	Yes	No	Yes	low
Verdugo-Paiva 2020	Yes	Yes	No	Yes	Yes	Yes	Yes	Yes	Yes	Yes	Yes	No	No	No	No	Yes	Very low
Verdejo 2020	Yes	Yes	No	Yes	Yes	Yes	Yes	Yes	Yes	Yes	Yes	Yes	Yes	No	No	Yes	low
Rada 2020	Yes	Yes	Yes	Yes	Yes	Yes	Yes	No	Yes	Yes	No meta-analysis	No meta-analysis	No	No	No meta-analysis	Yes	low
Maguire 2020	No	Yes	Yes	Yes	Yes	Yes	No	No	Partly Yes	No	No meta-analysis	No meta-analysis	No	No	No meta-analysis	Yes	Very low
John 2020	Yes	Yes	Yes	Yes	Yes	Yes	No	Yes	Yes	No	No meta-analysis	No meta-analysis	Yes	No	No meta-analysis	Yes	low
Baladia 2020	Yes	Yes	Yes	Yes	Yes	No	Yes	No	No	Yes	No meta-analysis	No meta-analysis	No	No	No meta-analysis	Yes	Very low

Appendix V：Characteristics of the included studies

Study	IF	Funding	Registration	Global cooperation	Number of institutions	Number of authors	PRISMA	Number of included studies
Wynants 2020	39.89	Yes	Yes	Yes	37	47	Yes	169
Siemieniuk 2020	39.89	Yes	No	Yes	33	57	Yes	196
Siemieniuk 2021	39.89	Yes	No	Yes	18	36	Yes	47
Bartoszko 2021	39.89	Yes	No	Yes	24	40	Yes	9
Allotey 2020	39.89	Yes	Yes	Yes	18	33	Yes	192
Allotey 2022	39.89	Yes	Yes	Yes	15	37	Yes	472
Wilt 2020	25.39	Yes	No	No	3	6	No	5
Schünemann 2020	25.39	Yes	Yes	Yes	16	50	Yes	143
Mackey 2020	25.39	No	Yes	No	2	8	Yes	83
Hernandez 2020	25.39	Yes	No	No	5	5	Yes	44
Helfand 2022	25.39	Yes	Yes	No	2	8	No	18
Juul 2020	11.06	Yes	Yes	Yes	8	14	Yes	82
Buitrago-Garcia 2020	11.06	Yes	No	No	2	8	Yes	94

Brümmer 2021	11.06	Yes	Yes	Yes	8	15	Yes	133
Schlesinger 2021	10.12	Yes	Yes	No	7	7	Yes	22
Griesel 2022	9.26	Yes	Yes	Yes	7	10	Yes	3
Webster 2021	9.26	Yes	Yes	Yes	6	6	No	1
Wagner 2021	9.26	Yes	Yes	No	8	12	Yes	11
Valk 2020	9.26	Yes	Yes	Yes	10	14	Yes	13
Stroehlein 2021	9.26	Yes	Yes	No	7	11	Yes	3
Salameh 2020	9.26	Yes	Yes	Yes	16	26	Yes	51
O'Byrne 2021	9.26	Yes	Yes	Yes	6	6	No	1
Mikolajewska 2021	9.26	Yes	Yes	Yes	12	12	Yes	4
Kreuzberger 2021	9.26	Yes	Yes	Yes	11	18	Yes	6
Ghosn 2021	9.26	Yes	Yes	Yes	15	24	No	12
Deeks 2020	9.26	Yes	Yes	Yes	9	17	Yes	57
Davidson 2022	9.26	Yes	Yes	Yes	15	22	No	6
Ansems 2021	9.26	Yes	Yes	No	6	10	Yes	5
Tleyjeh 2021	8.06	No	No	Yes	6	6	Yes	36

Qiu 2021	8.06	No	Yes	Yes	6	6	No	80
Langford 2020	8.06	Yes	Yes	No	8	8	No	24
Amer 2021	6.43	Yes	Yes	Yes	29	29	No	71
Harder 2021	6.30	No	Yes	No	1	8	Yes	30
Harder 2021	6.30	No	Yes	No	1	7	Yes	17
Santos 2020	6.11	Yes	Yes	No	4	8	Yes	183
Hussain 2021	5.81	Yes	No	Yes	4	7	Yes	6
Elvidge 2022	5.73	Yes	No	No	3	4	Yes	13
Centeno-Tablante 2020	5.69	Yes	Yes	Yes	4	10	Yes	37
Michelen 2021	5.50	Yes	Yes	Yes	14	20	Yes	39
Kirkham 2022	5.41	Yes	Yes	No	6	7	Yes	9
Zhang 2022	5.09	Yes	Yes	No	7	8	Yes	15
Gómez-Ochoa 2021	4.89	Yes	No	Yes	0	10	Yes	97
Dong 2021	4.83	Yes	No	No	3	5	Yes	44
Xu 2020	4.41	Yes	Yes	Yes	6	10	No	12

Bonardi 2022	4.36	Yes	Yes	Yes	17	23	Yes	9
Asiimwe 2021	4.33	Yes	Yes	No	2	6	Yes	9
Bell 2020	4.32	No	No	No	4	2	No	1
Soto-Cámara 2021	4.24	Yes	Yes	No	4	11	Yes	20
Yang 2021	3.63	No	Yes	No	6	7	Yes	45
Melo 2021	3.24	No	Yes	No	13	12	Yes	40
Korang 2022	3.24	Yes	Yes	Yes	13	20	Yes	46
Ceravolo 2020	2.87	No	Yes	No	7	10	No	25
Dzinamarira 2022	2.70	No	Yes	Yes	11	18	Yes	10
Silveira 2022	2.63	Yes	Yes	Yes	0	13	Yes	5
Cares-Marambio 2021	2.43	No	Yes	Yes	5	8	Yes	10

Bwire 2020	2.32	Yes	Yes	No	5	5	No	33
Rocha 2020	1.04	No	Yes	No	12	13	No	1
Verdugo-Paiva 2020	.00	Yes	Yes	Yes	4	4	Yes	12
Verdugo-Paiva 2020	.00	Yes	Yes	Yes	4	4	Yes	3
Verdejo 2020	.00	Yes	Yes	Yes	8	8	Yes	1
Rada 2020	.00	Yes	Yes	Yes	3	3	Yes	0
Maguire 2020	.00	Yes	Yes	Yes	16	29	Yes	728
John 2020	.00	Yes	Yes	No	13	18	Yes	78
Baladia 2020	.00	Yes	Yes	Yes	4	4	Yes	0

Appendix VI：List of the excluded studies

Study	Title	Reason for exclusion

Adrian 2020	A living mapping review for COVID-19 funded research projects: two year update	Living map
Van Thu 2021	Research response to coronavirus disease 2019 needed better coordination and collaboration: a living mapping of registered trials	Living map
Norton, A 2020	A living mapping review for COVID-19 funded research projects: nine-month update	Living map
Chiara 2022	The methodology of a "living" COVID-19 registry development in a clinical context	Methodological articles
Ralf 2022	Using COVID-19 Pandemic as a Prism: A Systematic Review of Methodological Approaches and the Quality of Empirical Studies on Physical Activity Behavior Change	Methodological articles
Guillaume 2021	Day-to-day discovery of preprint-publication links	Methodological articles
Kathryn 2022	Web-Based Software Tools for Systematic Literature Review in Medicine: Systematic Search and Feature Analysis	Methodological articles
Linda 2021	COVID-19 Technology-Enabled Living Systematic Reviews to Enhance Knowledge Translation	Methodological articles
Claire 2022	Methodological challenges for living systematic reviews conducted during the COVID-19 pandemic: A concept paper	Methodological articles
Christine 2020	Keeping Up With Emerging Evidence in (Almost) Real Time	Methodological articles

Sarah 2022	Development of Evidence-Based COVID-19 Management Guidelines for Local Context: The Methodological Challenges	Methodological articles
Stefano 2021	A systematic review that is ``rapid" and ``living": A specific answer to the COVID-19 pandemic	Methodological articles
Olivier 2022	Secondary electronic sources demonstrated very good sensitivity for identifying studies evaluating interventions for COVID-19	Methodological articles
Amir 2021	The Development of Living, Rapid Practice Points: Summary of Methods From the Scientific Medical Policy Committee of the American College of Physicians	Methodological articles
Gabriel 2020	Evidence synthesis relevant to COVID-19: a protocol for multiple systematic reviews and overviews of systematic reviews	Methodological articles
Juan R 2022	Artificial intelligence in COVID-19 evidence syntheses was underutilized, but impactful: a methodological study	Methodological articles
Francisca 2022	COVID-19 Living Overview of Evidence repository is highly comprehensive and can be used as a single source for COVID-19 studies	Methodological articles

Yiqiao 2022	Feasibility study for interactive reporting of network meta-analysis: experiences from the development of the MetaInsight COVID-19 app for stakeholder exploration, re-analysis and sensitivity analysis from living systematic reviews	Methodological articles
Dylan P 2021	Personal protective equipment for reducing the risk of COVID-19 infection among health care workers involved in emergency trauma surgery during the pandemic: An umbrella review	Overview of living systematic review
Julian 2022	Randomized trials on non-pharmaceutical interventions for COVID-19: a scoping review	Overview of living systematic review
Claire 2021	Methods and guidance on conducting, reporting, publishing and appraising living systematic reviews: a scoping review protocol	Overview of living systematic review
Malahat 2020	Therapeutic interventions for COVID-19: a living overview of reviews	Overview of living systematic review
Cuncun 2021	Coronavirus disease (COVID 2019): protocol for a living overview of systematic reviews	Overview of living systematic review
/	Update to living systematic review on prediction models for diagnosis and prognosis of covid-19	Previous version
/	Update to living systematic review on drug treatments for covid-19	Previous version
/	Update to living systematic review	Previous version
/	Update to living systematic review on prediction models for diagnosis and prognosis of covid-19	Previous version
/	Update to living systematic review on drug treatments for covid-19	Previous version

/	Update to living systematic review on covid-19 in pregnancy	Previous version
Amorim Dos Santos 2021	Oral Manifestations in Patients with COVID-19: A Living Systematic Review	Previous version
Elisa 2020	Systematic rapid living review on rehabilitation needs due to COVID-19: update to May 31st, 2020	Previous version
Elisa 2020	Rehabilitation and COVID-19: update of the rapid living systematic review by Cochrane Rehabilitation Field as of October 31st, 2021	Previous version
Elisa 2020	Rehabilitation and COVID-19: a rapid living systematic review 2020 by Cochrane Rehabilitation Field. Update as of September 30th, 2020	Previous version
Elisa 2020	Rehabilitation and COVID-19: update of the rapid living systematic review by Cochrane Rehabilitation Field as of February 28, 2021	Previous version
Anonymous 2020	Erratum: Update to living systematic review (BMJ (Clinical research ed.) (2020) 369 (m1328))	Previous version
Maria G 2020	Systematic rapid "living" review on rehabilitation needs due to COVID-19: update to March 31st, 2020	Previous version
Alessandro 2021	Rehabilitation and COVID-19: update of the rapid living systematic review by Cochrane Rehabilitation Field as of August 31st, 2021	Previous version
Alessandro 2020	Rehabilitation and COVID-19: the Cochrane Rehabilitation 2020 rapid living systematic review. Update as of August 31st, 2020	Previous version
Alessandro 2020	Systematic rapid living review on rehabilitation needs due to COVID-19: update as of April 30th, 2020	Previous version

Alessandro 2021	Rehabilitation and COVID-19: a rapid living systematic review by Cochrane Rehabilitation Field updated as of December 31st, 2020 and synthesis of the scientific literature of 2020	Previous version
Anjum S 2021	Major Update: Remdesivir for Adults With COVID-19 : A Living Systematic Review and Meta-analysis for the American College of Physicians Practice Points	Previous version
Francesco 2020	Rehabilitation and COVID-19: the Cochrane Rehabilitation 2020 rapid living systematic review. Update as of July 31st, 2020	Previous version
Francesco 2020	Rehabilitation and COVID-19: update of the rapid living systematic review by Cochrane Rehabilitation Field as of April 30, 2021	Previous version
Francesco 2021	Rehabilitation and COVID-19: a rapid living systematic review 2020 by Cochrane Rehabilitation Field. Update as of October 31st, 2020	Previous version
Francesco 2022	Rehabilitation and COVID-19: update of the rapid living systematic review by Cochrane Rehabilitation Field as of December 31st, 2021	Previous version
Amir 2022	Should Remdesivir Be Used for the Treatment of Patients With COVID-19? Rapid, Living Practice Points From the American College of Physicians (Version 2, Update Alert 3)	Previous version
Amir 2021	Update Alert 2: Should Remdesivir Be Used for the Treatment of Patients With COVID-19? Rapid, Living Practice Points From the American College of Physicians (Version 2)	Previous version
Amir 2021	Update Alert: Should Remdesivir Be Used for the Treatment of Patients With COVID-19? Rapid, Living Practice Points From the American College of Physicians (Version 2)	Previous version
Imad M 2021	Efficacy and safety of tocilizumab in COVID-19 patients: a living systematic review and meta-analysis	Previous version

Jie 2022	Coronavirus disease 2019 pandemic and pregnancy and neonatal outcomes in general population: A living systematic review and meta-analysis (updated Aug 14, 2021)	Previous version
George 2022	Prevalence of COVID-19 genomic variation in Africa: a living systematic review protocol	Protocol of living systematic review
Stefanie 2021	Pulmonary rehabilitation for COVID-19: A living systematic review protocol	Protocol of living systematic review
Giuliano 2020	Sexual transmission of SARS-CoV-2 virus and its role in the spread of COVID-19: A living systematic review protocol	Protocol of living systematic review
Tafadzwa 2021	Risk factors for COVID-19 among healthcare workers. A protocol for a systematic review and meta-analysis	Protocol of living systematic review
Benjamin P 2021	Repurposing existing medications for coronavirus disease 2019: protocol for a rapid and living systematic review	Protocol of living systematic review
Sophie 2020	Interventions for treatment of COVID-19: a protocol for a living systematic review with network meta-analysis including individual patient data (The LIVING Project)	Protocol of living systematic review
Aidan M 2021	Mesenchymal stromal cells as a therapeutic intervention for COVID-19: a living systematic review and meta-analysis protocol	Protocol of living systematic review
Steven Kwasi 2020	Vaccines to prevent COVID-19: a protocol for a living systematic review with network meta-analysis including individual patient data (The LIVING VACCINE Project)	Protocol of living systematic review

Brittany J 2020	A living systematic review protocol for COVID-19 clinical trial registrations	Protocol of living systematic review
Nicolás 2021	Angiotensin-converting-enzyme inhibitors and angiotensin II receptor blockers for COVID-19: A living systematic review of randomized clinical trials	Protocol of living systematic review
Melina 2020	What are the long-term symptoms and complications of COVID-19: a protocol for a living systematic review	Protocol of living systematic review
Lazar 2021	Epidemiology, clinical characteristics and treatment of critically ill patients with COVID-19): a protocol for a living systematic review	Protocol of living systematic review
Adam J 2020	The Sensitivity of Respiratory Tract Specimens for the Detection of SARS-CoV-2: A Protocol for a Living Systematic Review and Meta-Analysis	Protocol of living systematic review
María Belén 2021	Use of gloves for the prevention of COVID-19 in healthy population: A living systematic review protocol	Protocol of living systematic review
Jiyuan 2020	Prevalence of delirium, depression, anxiety, and post-traumatic stress disorder among COVID-19 patients: protocol for a living systematic review	Protocol of living systematic review
Abhinav 2020	Clinico-epidemiological characteristics of Kawasaki-like disease in paediatric patients with COVID-19: a protocol for rapid living systematic review	Protocol of living systematic review
Anup 2021	Impact of AYUSH interventions on COVID-19: a protocol for a living systematic review and meta-analysis	Protocol of living systematic review

Catalina 2020	Macrolides for the treatment of COVID-19: a living, systematic review	Protocol of living systematic review
Magnus 2020	Clinical manifestations, prevalence, risk factors, outcomes, transmission, diagnosis and treatment of COVID-19 in pregnancy and postpartum: a living systematic review protocol	Protocol of living systematic review
/	Correction: Interventions for treatment of COVID-19: A living systematic review with meta-analyses and trial sequential analyses (The LIVING Project)	Reply and correction
/	Correction: Accuracy of novel antigen rapid diagnostics for SARS-CoV-2: A living systematic review and meta-analysis	Reply and correction
/	RE: "COVID-19 IN HEALTH-CARE WORKERS: A LIVING SYSTEMATIC REVIEW AND META-ANALYSIS OF PREVALENCE, RISK FACTORS, CLINICAL CHARACTERISTICS, AND OUTCOMES"	Reply and correction
Ahmed 2021	Commentary: The Many Faces of COVID-19 at a Glance: A University Hospital Multidisciplinary Account From Milan, Italy	Reply and correction
Rohan 2022	Evaluating perinatal outcomes during a pandemic: A role for living systematic reviews	Reply and correction
Satheesh 2022	TASTE DISORDERS AND XEROSTOMIA ARE HIGHLY PREVALENT IN PATIENTS WITH COVID-19	Reply and correction
Shannon M 2021	In COVID-19, tocilizumab reduces all-cause mortality at 28 d	Reply and correction
S. A 2021	COVID-19 IN HEALTH-CARE WORKERS: A LIVING SYSTEMATIC REVIEW AND META-ANALYSIS OF PREVALENCE, RISK FACTORS, CLINICAL CHARACTERISTICS, AND OUTCOMES (vol 190, pg 161, 2021)	Reply and correction
Juul 2020	Interventions for treatment of COVID-19: A living systematic review with meta-analyses and trial sequential analyses (The LIVING Project) (vol 17, e1003293, 2020)	Reply and correction

Tarek 2021	Efficacy and safety of tocilizumab in COVID-19 patients: a living systematic review and meta-analysis - Author's reply	Reply and correction
David 2021	Re: 'Efficacy and safety of tocilizumab in COVID-19 patients: a living systematic review and meta-analysis' by Tleyjeh et al	Reply and correction
Catherine McLean 2020	Evidence based care for pregnant women with covid-19	Reply and correction
Xing 2021	Isolation and mental health: challenges and experiences from China	Reply and correction
Ruth S 2020	Access to evidence-based care for eating disorders during the COVID-19 crisis	Reply and correction
Agarwal, A 2021	Mucormycosis In Post Covid Patients: A Systematic Review	Unavailable full text
Ahdesmaki, O. 2021	PNS7 Ongoing SLRs When Considering HTA Submissions: Rewards and Avoidable Pitfalls	Unavailable full text
Bach-Mortensen, A 2021	OWNERSHIP AND COVID-19 IN CARE HOMES FOR OLDER PEOPLE: A LIVING SYSTEMATIC REVIEW OF OUTBREAKS, INFECTIONS, AND MORTALITY	Unavailable full text
Daniyal Mehmood 2021	Facilitating Undergraduate Research Using a Case Study Approach - A Race to Normalcy During the COVID-19	Unavailable full text
Charbel 2021	MM-450: Anti-Thrombotic Therapy for Ambulatory Patients with Multiple Myeloma Receiving Immunomodulatory Agents: A Systematic Review	Unavailable full text
Elizabeth 2021	COVID-19 PNEUMONIA COMPLICATED BY CMV PNEUMONITIS FOLLOWING TREATMENT WITH AN IL-6 INHIBITOR	Unavailable full text

Irbaz Bin 2019	Living systematic reviews: A novel mechanism for improving efficiency and quality of evidence synthesis in oncology	Unavailable full text
Irbaz Bin 2019	A living systematic review of immune checkpoint inhibitors in cancer patients: A novel platform for evidence synthesis in oncology	Unavailable full text
Spyridis, N 2021	Outcomes of Patients with Hematologic Malignancies and COVID-19: Perspective of a Large Hematology Center in Greece	Unavailable full text
